# The *RAD51* paralogue *HvXRCC2* affects meiosis and recombination in barley

**DOI:** 10.1093/jxb/eraf489

**Published:** 2025-12-18

**Authors:** Isabelle Colas, Malcolm Macaulay, Mikel Arrieta, Miriam Schreiber, Jamie Orr, Amritpal Sandhu, Anu Augustine, Małgorzata Targońska-Karasek, Susan J Armstrong, Robbie Waugh, Luke Ramsay

**Affiliations:** Cell and Molecular Sciences, The James Hutton Institute, Invergowrie, Dundee DD2 5DA, UK; Cell and Molecular Sciences, The James Hutton Institute, Invergowrie, Dundee DD2 5DA, UK; Cell and Molecular Sciences, The James Hutton Institute, Invergowrie, Dundee DD2 5DA, UK; Information and Computational Sciences, The James Hutton Institute, Invergowrie, Dundee DD2 5DA, UK; Cell and Molecular Sciences, The James Hutton Institute, Invergowrie, Dundee DD2 5DA, UK; School of Biosciences, University of Birmingham, Edgbaston, Birmingham B15 2TT, UK; Department of Biotechnology and Microbiology, Kannur University, Dr. Janakiammal Campus, Palayad, Thalasserry, Kerala 670661, India; Polish Academy of Sciences Botanical Garden, Center for Biological Diversity Conservation in Powsin, 2 Prawdziwka Street, Warszawa, 02-973, Poland; Polish Academy of Sciences Botanical Garden, Center for Biological Diversity Conservation in Powsin, 2 Prawdziwka Street, Warszawa, 02-973, Poland; Cell and Molecular Sciences, The James Hutton Institute, Invergowrie, Dundee DD2 5DA, UK; Division of Plant Sciences, University of Dundee at The James Hutton Institute, Invergowrie, Dundee DD2 5DA, UK; Cell and Molecular Sciences, The James Hutton Institute, Invergowrie, Dundee DD2 5DA, UK; Cardiff University, UK

**Keywords:** Barley, crossover, desynaptic, immunocytology, meiosis, recombination, XRCC2

## Abstract

Using a positional candidate-gene approach, we show that semi-sterile *desynaptic8* mutants are associated with deleterious variants of the barley homolog of *XRCC2* (*X-Ray Repair Cross Complementing 2*). In barley *XRCC2* mutants, the initial meiotic progression is normal, albeit with a small delay in initiation, with completion of synapsis. However, the absence of *HvXRCC2* subsequently leads to a dramatic reduction in the number of crossovers, chromosome mis-segregation, and infertility, suggesting that *HvXRCC2* plays a major role in recombination. This mutant phenotype is congruent with that reported in mammalian studies but contrasts with the *XRCC2* mutant in Arabidopsis which is fertile, exhibits normal chromosome pairing and correct chromosome segregation, and is associated with an increased rate of crossovers. This observation indicates that the *XRCC2* mutant phenotype in Arabidopsis is not representative of all plants and that *XRCC2* is not a good candidate for the modulation of recombination in barley.

## Teaser

Mutants of the barley homolog of *XRCC2* exhibit delays in replication leading to defective meiosis, altered RAD51 orthologue behaviour, and a significant reduction in the number of crossovers, as in canonical mammalian *XRCC2* mutants but unlike those in Arabidopsis.

## Introduction

Meiosis is the specialized process required for sexual reproduction that involves two cell divisions after a single round of DNA replication that results in gametes with half the genetic complement of the parent (Mercier *et al*., 2014). During meiosis I, two major interlinked events occur simultaneously: chromosome synapsis and homologous recombination (HR). Synapsis aligns the homologous chromosomes along their entire length, providing the necessary proximity to promote effective recombination (Zickler, 2006). HR is essential for chromosome segregation during the first division of meiosis but also for the exchange of genetic material via crossovers that create unique allelic combinations in each of the four haploid gametes (Zickler, 2006; Wang and Copenhaver, 2018). During meiosis, HR starts with SPO11-catalysed DNA double-strand breaks, which are subsequently resected to generate a 3′ single-stranded DNA end (reviewed in Mercier *et al*., 2014). The 3′ ends are then captured by the RecA-like proteins RAD51 and DMC1 (meiosis specific) to promote strand invasion and repair of double-strand breaks by the homologous DNA template (Brown and Bishop, 2014). During meiosis, this strand invasion is essential for the formation of a ‘D-loop’, which leads to a crossover when repaired via the double Holliday junction pathway (Daley *et al*., 2014; Wang and Copenhaver, 2018).

Five *RAD51* paralogues (*RAD51B*, *RAD51C*, *RAD51D*, *XRCC2*, and *XRCC3*) have been found in both mammals and plants (Da Ines *et al*., 2012; Da Ines *et al*., 2013a; Sullivan and Bernstein, 2018). These form two distinct complexes, BCDX2 (RAD51B-RAD51C-RAD51D-XRCC2) and CX3 (RAD51C-XRCC3), both of which are involved in meiotic and somatic recombination, DNA repair, and chromosome stability (Masson *et al*., 2001; Chun *et al*., 2013; Sullivan and Bernstein, 2018).

Effective null mutation of *XRCC2* leads to a delay in (but not an absence of) RAD51 foci formation in hamster cells (Liu, 2002), and numerous studies have shown that various *XRCC2* mutants are hypersensitive to DNA cross-linking agents such as mitomycin C, cisplatin, aldehydes, tirapazamine, and temozolomide, and that DNA replication fork dynamics are disturbed in these mutants (Liu *et al*., 1998; Liu, 2002; Liu and Lim, 2005; Wang *et al*., 2014; Saxena *et al*., 2018). In Arabidopsis and more recently in Drosophila, *XRCC2* has also been shown to be important for somatic recombination and DNA repair under genotoxic stress while having a minor (or no evident) role during meiosis (Bleuyard *et al*., 2005; Da Ines *et al*., 2013a; Wang *et al*., 2014; Bayer *et al*., 2020). This contrasts with mammalian studies where *xrcc2* effective null mutations cause chromosome mis-segregation (Cui *et al*., 1999; Griffin *et al*., 2000; Mozdarani *et al*., 2001), developmental defects (Adam *et al*., 2007), and meiotic arrest and infertility (Griffin *et al*., 2000). Infertility also results from a point mutation (41T>C) in human and mouse *XRCC2* (Yang *et al*., 2018). Female Drosophila *xrcc2* mutants, on the other hand, are fertile and have normal development, which has been attributed to a postulated redundancy of the proteins XRCC2 and XRCC3 (Bayer *et al*., 2020). In Arabidopsis, *xrcc3* null mutants are defective for meiosis, but *xrcc2* null mutants are fertile and exhibit normal chromosome pairing, synapsis, and correct chromosome segregation (Bleuyard and White, 2004; Bleuyard *et al*., 2005; Da Ines *et al*., 2013a). Interestingly, the *xrcc2* null mutant in Arabidopsis was also reported to be associated with an increased rate of crossovers and recombination (Da Ines *et al*., 2013a).

There is considerable interest in mutants in genes such as *FANCM*, *RecQ4*, and *Hei10*, which increase crossover number in Arabidopsis, due to their potential application in crop breeding (Mieulet *et al*., 2018; Arrieta *et al*., 2021). The potential to modulate recombination is particularly attractive in large-genome cereals such as barley, where crossover distribution patterns are strongly skewed towards the ends of the chromosomes, limiting the potential recombination of large sections of the genome (Kunzel and Waugh, 2002; Mascher *et al*., 2017; Lambing and Heckmann, 2018). The behaviour of *XRCC2* mutants in plant species other than Arabidopsis is thus of interest given potential breeding and genetic applications.

**Fig. 1.**
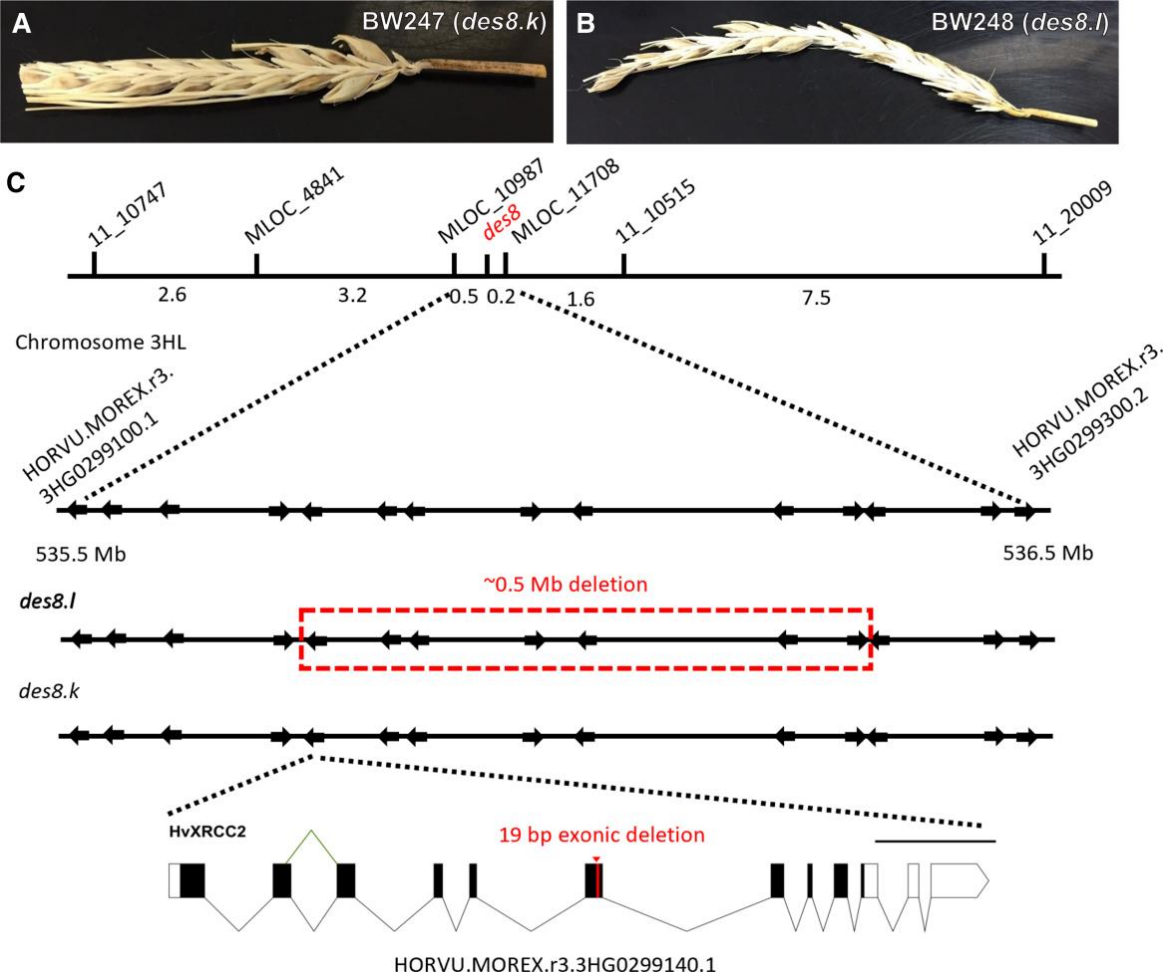
Mapping of *des8*. (A, B) *desynaptic 8* mutants exhibit a semi-sterile phenotype. (C) The *des8* region was initially delineated between two SNP markers (11_20659 and 11_10515) on chromosome 3H and then fine mapped on an extended F_2_ population to an <0.5 cM region between two markers (MLOC_10907 and MLOC_11708) corresponding to a region between HORVU.MOREX.r3.3HG0299100.1 and HORVU.MOREX.r3.3HG0299300.2. *des8.l* in BW248 is a large deletion of 0.5 Mb, whereas *des8.k* in BW247 has a small 19 bp exonic deletion in *HvXRCC2* (HORVU.MOREX.r3.3HG0299140).

*desynaptic8* (*des8*) was first described by Hernandez-Soriano (1973) in spontaneous semi-sterile mutants found in the barley cultivar Betzes (Hockett and Eslick, 1969). Two alleles, *des8.k* and *des8.l*, are known, both exhibiting similarly perturbed meiosis with univalents present at Metaphase I, although the associated semi-sterility is reported to be stronger in *des8.l* (Fig. 1A, B) (Hernandez-Soriano, 1973; Lundqvist *et al*., 1997). Here, using a positional candidate-gene approach, we show that *des8* mutants (Hernandes-Soriano, 1973; Druka *et al*., 2010) are associated with deletions in or of the barley homolog of *XRCC2*, enabling us to compare the meiotic phenotype and effect on recombination of the mutants with that in the model plant Arabidopsis.

## Materials and methods

### Plant and material preparation

The cultivar Bowman [wild type (WT)] and its near-isogenic lines (NILs) BW247 (BC_2_F_3_; *des8.k*) and BW248 (BC_4_F_3_; *des8.l*) were grown under 16 h of light at 18–20 °C and 8 h of dark at 16 °C in general-purpose mix compost containing peat, sand, limestone, perlite, Celcote, Osmocote®, and Exemptor®, and were used in all the experiments. After 7 weeks from sowing, anthers were checked for meiotic stage using aceto-carmine spreads and fixed for cytology. For mapping and recombination studies, F_2_ and derived F_3_ populations resulting from BW247 (*des8.k*) × cv. Morex, BW248 (*des8.l*) × cv. Barke, or BW248 (*des8.l*) × cv. Morex were grown in a glasshouse, and young leaf tissue from ∼2-week-old seedlings were collected into 96-well plates for DNA extraction as described previously (Arrieta *et al*., 2021). Plants were grown to maturity to allow the assessment of fertility and harvest.

### Mapping and sequencing

Initial genetic mapping was based on 94 F_2_ individuals derived from a cross between BW248 (*des8.l*) and the cultivar Barke (WT) using segregation of the semi-sterile phenotype of *des8.l* as a Mendelian trait. Genotyping was carried out using an Illumina 384 SNP BeadXpress platform as described previously (Colas *et al*., 2016). Subsequent mapping was carried out in 920 F_2_ individuals derived from a BW428 (*des8.l*) × Morex cross via the iterative development of custom KASPar© SNP assays (KBioscience). These diagnostic KASP assays were designed as previously described (Arrieta *et al*., 2021) from alignments of genic sequences known to map in the genomic interval mined for polymorphism between BW248 and Morex. Initial genetic mapping of the BeadXpress data of the F_2_ segregating population used segregation of the semi-sterile phenotype of *des8.l* as a Mendelian trait. JoinMap 4.0 (Kyazma) software was used to assign marker loci to linkage groups, and two rounds of regression mapping was used to order the loci within groups. For fine mapping, iterative rounds of single nucleotide polymorphism (SNP) marker development were utilized on progressively smaller subsets of the F_2_ population shown to contain informative recombination events. Sequencing of the coding domains of high-confidence gene models was carried out by standard Sanger sequencing, as was confirmation of the BW247 (*des8.k*) cDNA sequence, as previously described (Colas *et al*., 2016).

### Recombination study

The effect of *des8.l* on the recombination landscape was studied using the segregation of SNP loci in F_3_ families derived from specific F_2_ individuals from a BW248 (*des8.l*) × Barke cross that were homozygous for either the WT or the mutant allele at *HvXRCC2*. Genotyping was carried out using the Barley 50K iSelect SNP array (Bayer *et al*., 2017) as previously described (Arrieta *et al*., 2021). Subsequent 50K data handling and recombination analysis were carried out using custom R scripts (see Data availability). Briefly, monomorphic markers between the BW248 and Barke genotypes were removed. F_3_ marker calls were converted to a numeric matrix representing a match to either parental genotype (0 and 2) or a heterozygous call (1). A sliding window was used to adjust genotyping calls to the median within a 20-marker window to account for poorly mapped markers. Outlier markers and F_3_ individuals containing a large number of expected transitions (>95% quantile and >98.5% quantile, respectively) along the chromosome were removed. Informative (F_2_ heterozygous) and uninformative (F_2_ homozygous) regions were determined by the presence or absence of large monomorphic blocks matching either genotype in the original cross in F_3_ individuals grouped by F_2_ parent. Crossovers were counted at the junction between three continuous matches/mismatches to the F_2_ parental genotype in each direction. The recombination frequency for each marker for each segregating phenotype was calculated as the total number of crossovers observed divided by the number of observations for this marker within an F_2_ heterozygous region.

### Bleomycin/mitomycin C DNA damage repair assay

Seeds of Bowman, BW247, and BW248 were sterilized with 70% ethanol for 30 s and rinsed three times for 5 min with sterile water. Seeds were subsequently surface sterilized with 7% sodium hypochlorite for 4 min and then rinsed three times for 5 min, followed by a wash for 1 h in sterile water. Seeds were grown on 0.5% DUCHEFA Phyto agar (Melford Laboratories Ltd, UK) for the control, and also with the addition of mitomycin C (Abcam, UK), 10 mg l^–1^ or bleomycin (Cambridge Bioscience, UK) 50 mg l^–1^, respectively, for 7 d at 21 °C, and the root length was measured.

### DNA *in situ* hybridization

Anthers were fixed in ethanol:acetic acid (3:1) for 24 h and then stored in 70% ethanol at 4 °C until use. Slide preparation and DNA *in situ* hybridizations were performed as previously described (Higgins *et al*., 2012; Colas *et al*., 2016) using digoxigenin-labelled 5S rDNA and biotin-labelled 45S rDNA, barley sub-telomeric HvT01 labelled with FITC-dUTP, and centromeric BAC7 labelled with biotin-dUTP (Jasencakova *et al*., 2001).

### Immunocytology

Anthers were collected in working buffer (1× PBS/0.5% Triton^TM^ X-100/0.5% Tween-20) until fixation in 4% formaldehyde in working buffer for 20 min. Following two washes of 10 min in 1× PBS, the anthers were tapped to release the meiocytes. The meiocyte suspensions (30 µl) were transferred on to a poly-L-lysine coated slide (Polysine®) and left to air dry at room temperature. Slides were first blocked for 20 min in 5% goat/donkey serum prepared in working buffer, followed by incubation in the primary antibody solution, which consisted of one or multiple antibodies diluted in blocking solution in a wet chamber for 1 h at room temperature. This was followed by 24–36 h incubation at 4 °C. The following antibodies were used: rabbit TaASY1 (1:2000), rat HvZYP1 (1:500), guinea pig HvDMC1 (1:200), rat HvRAD51 (1:200), rabbit HvMLH3 (1:500), and rabbit H3K27me3; (Baker *et al*., 2015; Colas *et al*., 2016; Colas *et al*., 2019). HvRAD51 polyclonal antibody was developed for this study by immunizing a rat with two custom-designed peptides as per the supplier’s protocol (Dundee Cell Product, UK). Slides were warmed for 30 min to 1 h at room temperature before washing for 15 min in 1× PBS and then incubating for up to 2 h at room temperature in a secondary antibody solution. Secondary antibodies consisted of a mixture of anti-rabbit Alexa Fluor® (488, 568, or 647), anti-rat Alexa Fluor® (568, 488, or 647) and/or anti-guinea pig Alexa Fluor® (568 or 488) (Invitrogen^TM^) diluted in blocking solution (1:300). Slides were washed in 1× PBS, counterstained for 10 min in a wet chamber with Hoechst 33342 (2 µg ml^–1^, Invitrogen^TM^), and mounted in ProLong™ Gold antifade mounting medium (Thermo Fisher Scientific).

### Time course

We used a Click-iT™ EdU (5-ethynyl-2′-deoxyuridine) Alexa Fluor™ 647HCS assay kit (Invitrogen^TM^) as previously described (Colas *et al*., 2016). Fixed anthers were prepared for immunodetection as described above with TaASY1 and HvDMC1 or HvRAD51, immediately followed by EdU detection as per the supplier’s protocol. EdU was detected after 45 min incubation instead of the 30 min described in the supplied protocol. Slides were counterstained with Hoechst 33342 (2 µg ml^–1^, Invitrogen^TM^) and mounted in ProLong™ Gold Antifade mounting medium (Thermo Fisher Scientific).

### Microscopy and imaging

Three-dimensional (3D) confocal stack images (512×512, 12 bits) were acquired with a LSM 710 confocal microscope (Zeiss) using laser light at 405, 488, 561, and 633 nm sequentially. Projections of 3D pictures and light brightness/contrast adjustment were performed with Imaris 9.5.1 (Bitplane). 3D structured illumination microscopy (3D-SIM) images were acquired on a DeltaVision OMX Blaze (GE Healthcare) microscope using laser light at 405, 488, and 564 nm, as previously described (Colas *et al*., 2016). Image analyses were performed with Imaris 9.5 as previously described (Colas *et al*., 2016) and Imaris image deconvolution was performed using ClearView 9.5.

## Results

### Genetic mapping of *des8*

Both *des8.k* and *des8.l* have been used as donor parents in an extensive backcrossing programme using the cultivar Bowman as the recurrent parent, generating the NILs BW247 (Bc_2_F_3_) and BW248 (Bc_4_F_3_), respectively (Druka *et al*., 2011). Initial genotyping of BW247 and BW248 with 3072 SNPs (BOPA1 and BOPA2) (Close *et al*., 2009) allowed comparisons of the NILs with the donor and recurrent parent cultivars and indicated the presence of introgressions on 3HL and 5HL for BW247 and 3H and 6HL in BW248 (Druka *et al*., 2011).

We initially mapped *des8.l* genetically as a Mendelian trait based on segregation of its semi-sterile phenotype in 94 F_2_ individuals derived from a cross between BW248 (*des8.l*) and the WT cv. Barke (Fig. 1B). The use of an Illumina 384 SNP BeadXpress platform enabled *des8* to be mapped to the long arm of chromosome 3H between SNPs 11_10747 and 11_20009, which map within the genes HORVU.MOREX.r3.3HG0297410.1 and HORVU.MOREX.r3.3HG0302040.1, respectively. This position overlaps with the introgressions observed on chromosome 3H in BW247 and BW248 (Druka *et al*., 2011). Given a lack of polymorphism in the cross with Barke, subsequent mapping was carried out using 920 F_2_ individuals derived from a BW248 (*des8.l*) × cv. Morex cross. The iterative development of custom KASPar© SNP assays (KBioscience) derived from alignments of genic sequences known to map in this interval were mined for polymorphism between BW248 and Morex, and these were used to delineate the interval containing *des8.l* to that bounded by MLOC_10987 (HORVU.MOREX.r3.3HG0299100.1) and MLOC_11708 (HORVU.MOREX.r3.3HG0299300.2) (Fig. 1C). The interval containing *des8.l* was thus mapped to within a single 1.37 Mb bacterial artificial chromosome (BAC) contig (contig_1227) (https://apex.ipk-gatersleben.de/apex/f?p=284:10::::::) to a region containing 14 high-confidence annotated genes (Fig. 1C). Using the sequence information within BAC contig_1227, primers were designed across the coding domains of the genes bounded by flanking SNPs. This indicated that seven high-confidence genes were missing in BW248 (*des8.l*) relative to Morex (Supplementary Table S1), a deletion of over 474 kb (Fig. 1C). Resequencing these seven genes in BW247 (*des8.k*) found that all were identical to the original *des8.k* mutant donor Betzes except for HORVU.MOREX.r3.3HG0299140.1, which carried a 19 bp exonic deletion (Fig. 1C). These results indicate that *des8* mutants are caused by mutations involving HORVU.MOREX.r3.3HG0299140.1, the barley homolog of *X-Ray Repair Cross Complementing 2* (*XRCC2*).

*HvXRCC2* consists of 12 exons, contains two 3′UTR introns (Fig. 1C), and encodes a protein of 347 amino acids that shows a high level of conservation with other known XRCC2 proteins (Supplementary Fig. S1) that include the P-Loop_NTPase superfamily domain, characterized by the Walker A and Walker B motifs (Supplementary Fig. S2). The 19 bp deletion in the *des8.k* allele (BW247) (Supplementary Fig. S3) was confirmed by cDNA sequencing and is in the sixth exon of *HvXRCC2*. It is predicted to produce a truncated protein of 262 amino acids (Fig. 1C; Supplementary Fig. S3). A gene expression atlas across 16 tissues of cv. Morex (Rapazote-Flores *et al*., 2019) indicated that *HvXRCC2* is expressed in all tissues but highly expressed in developing inflorescence (Supplementary Fig. S4A). In addition, a meiotic-specific expression dataset from the cultivar Golden Promise indicated that *HvXRCC2* was highly expressed in meiocytes during G2 and leptotene/zygotene stages, but less expressed from pachytene onward (Barakate *et al*., 2021) (Supplementary Fig. S4B). *HvXRCC2* (HORVU.MOREX.r3.3HG0299140) exists as two main isoforms (Barakate *et al*., 2021): BAnTr.GP.3HG012678.1, whose expression appears to be meiosis specific, and BAnTr.GP.3HG012678.2, which has an alternative 5′ splice site at the end of exon 2 that leads to the loss of three nucleotides and consequently a single glycine in the protein, and which is expressed at a lower level than BAnTr.GP.3HG012678.1 (Supplementary Fig. S4B). The presence of these two isoforms is supported in the BaRTv1.0 barley transcript dataset visualized via the EoRNA database (Milne *et al*., 2021). The latter transcript (BaRT1_0u23042.002) potentially has a more mitotic role with stronger expression in apical meristem libraries, although interpretation is complicated as all *HvXRCC2* transcripts in EoRNA are 5′ truncated, possibly due to issues in earlier genome coverage (Mascher *et al*., 2017) and resultant gene models (e.g. HORVU3Hr1G082620.28).

### Investigation of the role of *HvXRCC2* in somatic cells in barley

Given the apparent ubiquitous expression of XRCC2 in the EoRNA barley expression database (https://ics.hutton.ac.uk/eorna/index.html) and the report of an effect on both mitosis and meiosis in Arabidopsis (Da Ines *et al*., 2013a; Wang *et al*., 2014) the *des8* mutants were investigated for a possible somatic role. We tested the response of the *des8* lines to the application of genotoxic agents using the DNA cross-linking agent mitomycin C and the radiomimetic agent bleomycin (Liu and Lim, 2005; Da Ines *et al*., 2013a; Wang *et al*., 2014). The root lengths of the NILs containing *des8* alleles were not significantly different from those of the Bowman WT in the absence of genotoxic agents, and the presence of genotoxic agents had a significant effect on root length (*P*<0.005, Welch’s *t*-test) in all lines. No significant difference was found between lines when grown in the presence of bleomycin; however, in the presence of mitomycin C, BW248 (*des8.l*) had significantly shorter roots than Bowman WT (*P*<0.05, Welch’s *t*-test), although BW427 (*des8.k*) did not (Supplementary Fig. S5; Supplementary Table S2).

### *des8.k* and *des8.l* undergo complete synapsis despite a short delay at the beginning of the synaptonemal complex formation

**Fig. 2.**
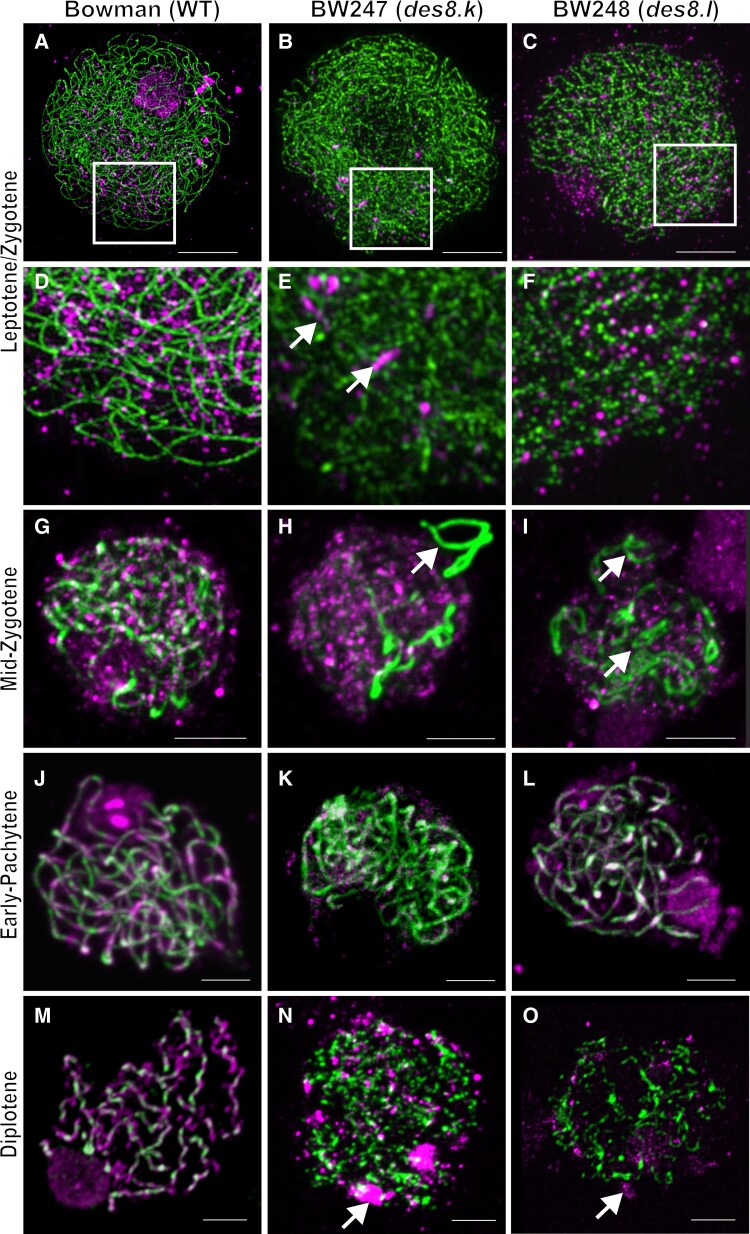
Comparison of synapsis in Bowman, BW247, and BW248. Synapsis of all genotypes was tracked by immunolabelling of ASY1 (green) and ZYP1 (magenta) at leptotene/zygotene (A–F), mid-zygotene (G–I), pachytene (J–L), and diplotene (M–O). Arrows in (E) indicate ZYP1 stretches, arrows in (H, I) indicate un-synapsed ASY1-labelled chromosomes, and arrows in (N, O) indicate ZYP1 aggregates. Scale bars=5 µm.

Using TaASY1 and HvZYP1 antibodies, the progression of synapsis was followed in Bowman (WT), BW247 (*des8.k*), and BW248 (*des8.l*) meiotic cells (Fig. 2). In all three lines, ZYP1 started to polymerize at one side of the nucleus during leptotene/zygotene (Fig. 2A–F). ZYP1 polymerized in between the homologous chromosomes to form continuous ZYP1 filaments in the WT (Fig. 2D), but not in BW247 and BW248, where ZYP1 formed short discontinuous stretches (Fig. 2E, F) at the same stage. Later, during zygotene, ZYP1 polymerized along the chromosomes in all genotypes, but the appearance of un-synapsed ASY1 loops was frequent in BW247 and BW248 (Fig. 2H, I, arrows), suggesting that the progression of synapsis was subject to some delay in the mutants (Colas *et al*., 2016). By pachytene, synapsis was completed, as shown by the full ZYP1 polymerization and thick chromosome axes in all genotypes (Fig. 2J–L). All three genotypes reached diplotene, exhibiting tinsel chromosome structures (Colas *et al*., 2017) (Fig. 2M–O), although ZYP1 aggregates were visible in both mutants but not in the WT (Fig. 2N, O, arrows).

### Synapsis delay affects the behaviour of RAD51 and DMC1 in both *des8.k* and *des8.l*

**Fig. 3.**
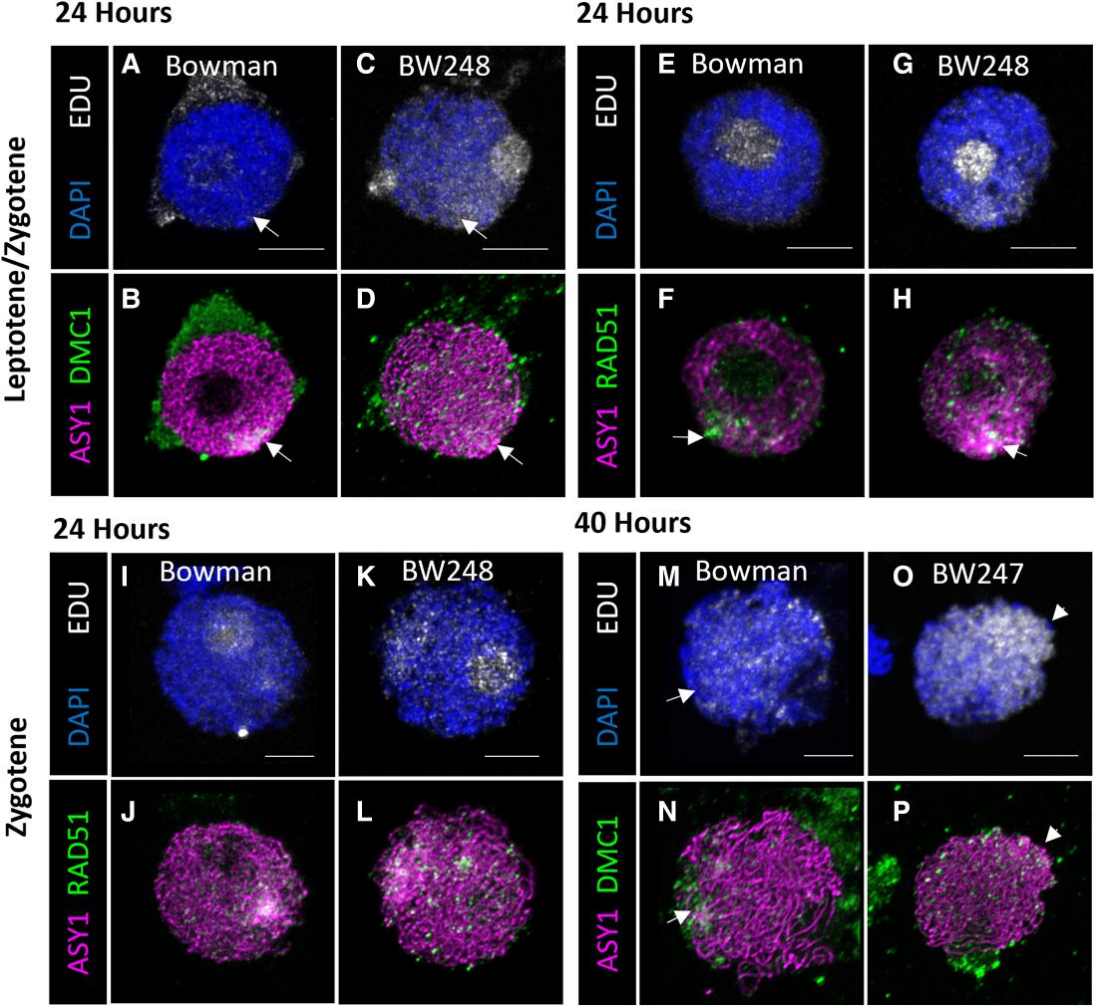
Time course analysis of early recombination events. Time course in Bowman and *des8* mutants using immunolabelling for EdU (white), ASY1 (magenta), and HvDMC1 (green) or HvRAD51 (green). (A–H) Labelling at 24 h with HvDMC1 (B, D) and HvRAD51 (F, H) in cells at leptotene/zygotene in Bowman (A, B, E, F) and BW248 (C, D, G, H). (I–L) Labelling at 24 h with HvRAD51 in cells at zygotene in Bowman (I, J) and BW248 (K, L). (M–P) Labelling at 40 h with HvDMC1 in cells at zygotene in Bowman (M, N) and BW247 (O, P). Arrows indicate the position where recombination starts, polarized in telomeric regions. In (A–D, N, P). residual cytoplasm can be seen outside the nucleus. Scale bars=5 µm.

Due to the presence of the atypical short ZYP1 stretches in BW247 and BW248 during leptotene/early zygotene, and the known involvement of XRCC2 in replication fork progression (Liu and Lim, 2005; Saxena *et al*., 2018), we hypothesized that entry into meiosis could be compromised in the *des8* mutants. We conducted a time-course experiment to monitor the start of synapsis by injecting 1–1.5 cm long spikes with an EdU solution for 2 h and collecting the injected spikes at 24 h and 40 h, as previously described (Colas *et al*., 2016). Cells at leptotene/zygotene were checked for EdU, ASY1 axis, and the early recombination proteins HvDMC1 and HvRAD51 (Fig. 3). As cells start meiosis only when replication is completed, any EdU labelling indicated that the cells had been replicating in the presence of EdU within the incubation period.

After 24 h, leptotene/zygotene cells in Bowman were not EdU labelled, indicating that replication was completed in these cells prior to EdU injection (Fig. 3A, E). At this stage, ASY1 axes are fully formed and both HvDMC1 and HvRAD51 foci congregate towards a brighter ASY1 region that resembles the telomere cluster (Fig. 3B, F, arrows). BW248 cells at the same stage were partially labelled with EdU, indicating that some replication had occurred within the past 24 h (Fig. 3C, G). As in the WT, ASY1 axes appeared fully formed, and both HvDMC1 and HvRAD51 foci congregated towards one side of the nucleus (Fig. 3D, H, arrows). HvDMC1 foci were, however, more dispersed within BW248 nuclei compared with the WT (Fig. 3D).

In WT cells that were at zygotene at 24 h, faint EdU labelling was evident, with a brighter spot at one side of the nucleus (Fig. 3I), indicating that these cells had recently finished late replication. ASY1 axes were linear and RAD51 foci clustered to the EdU-labelled side of the nuclei (Fig. 3J). We observed a similar result in BW248, but with stronger EdU labelling than in the WT (Fig. 3K), indicating that replication was slightly longer in the mutant. RAD51 foci were also more dispersed within the nuclei compared with the WT (Fig. 3L).

At 40 h, most labelled WT nuclei were at zygotene and were homogenously labelled with EdU (Fig. 3M). The WT ASY1 axes were thick but were beginning to be less evident in stretches where synapsis had been initiated. Similarly, HvDMC1 foci were still concentrated to one side of the nucleus but foci were showing signs of starting to diffuse (Fig. 3N). BW247 nuclei were also labelled with EdU but the signal intensity was much brighter than in the WT, with an even stronger signal at one side of the nucleus (Fig. 3O). Similar to the WT, HvDMC1 foci were still concentrated at one side of the nucleus in BW247, although starting to diffuse (Fig. 3P). However, the clusters of HvDMC1 foci in BW247 and BW248 were not as tight as in the WT at any time point (Fig. 3D, P).

**Fig. 4.**
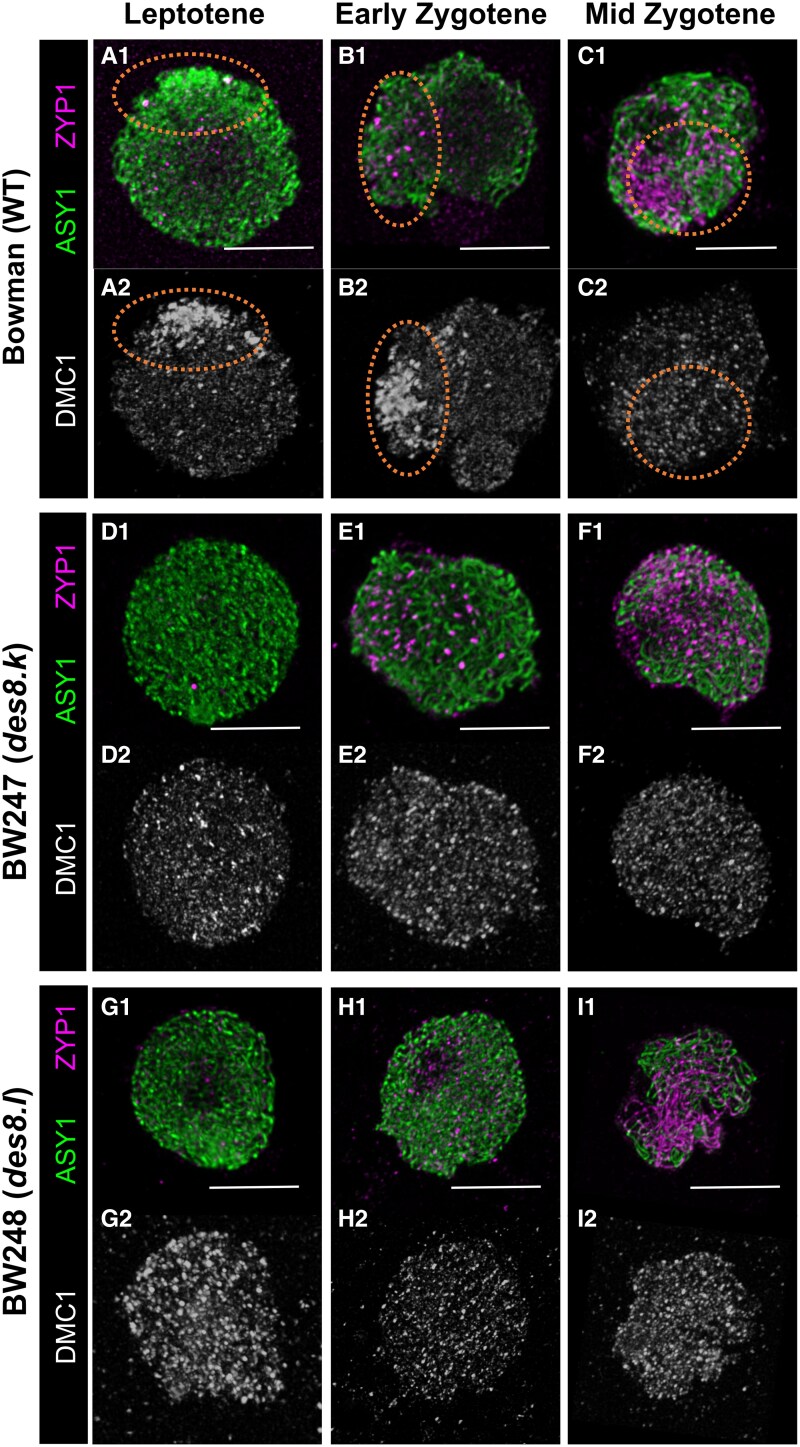
DMC1 behaviour during synapsis. Immunolabelling of Bowman (A–C), BW247 (D–F), and BW248 (G–I) with ASY1 (green), ZYP1 (magenta), and DMC1 (white). Orange ellipses indicate the telomere area where DMC1 foci normally start loading. Scale bars=5 µm.

**Fig. 5.**
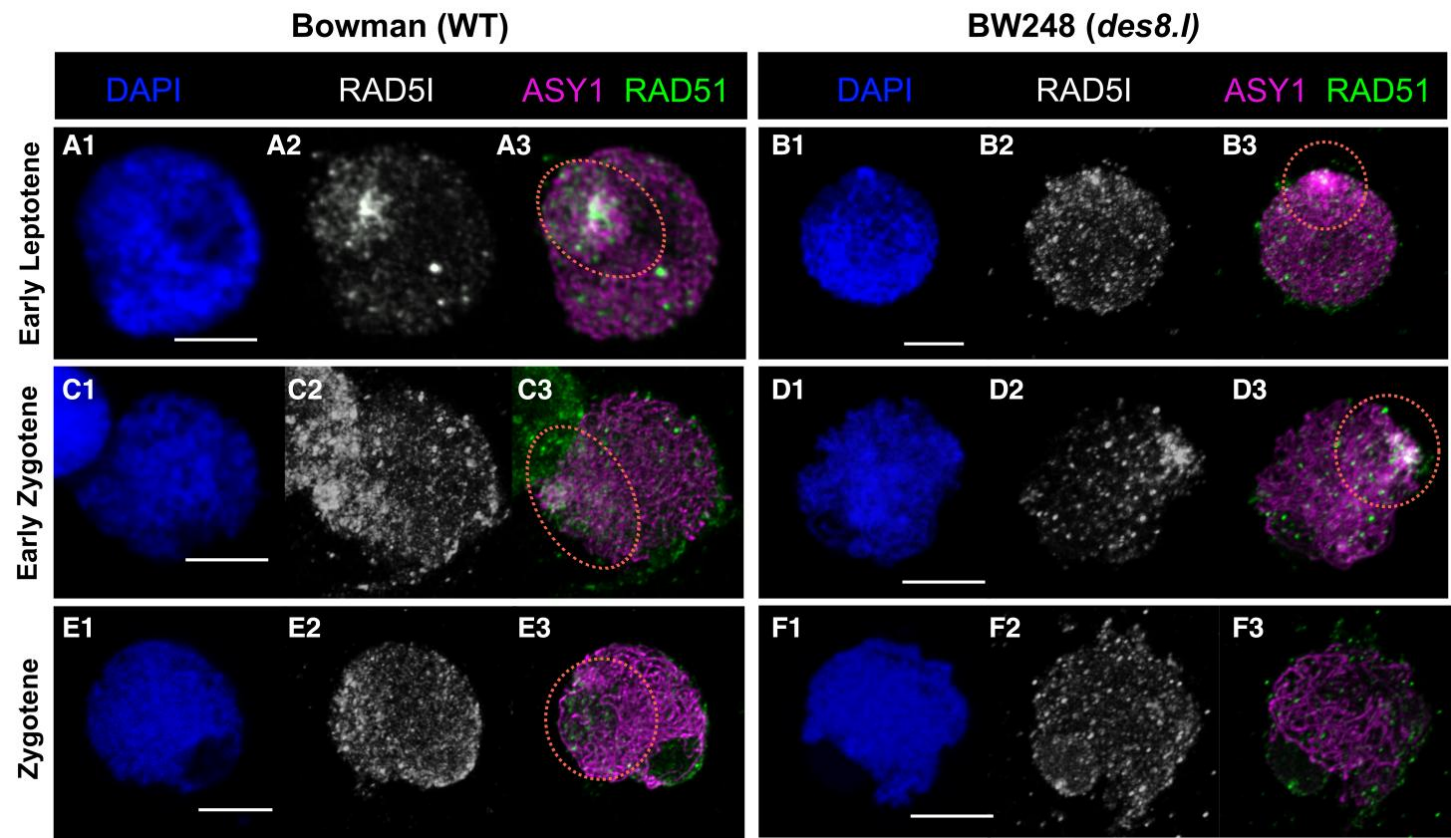
RAD51 behaviour during synapsis. Immunolabelling of Bowman (A, C, E) and BW248 (B, C, F) with ASY1 (magenta) and RAD51 (green/white). Orange ellipses indicate the telomere area where RAD51 foci normally start loading. Scale bars=5 µm.

The time-course experiment suggested that replication was affected, taking longer in both mutants, with more EdU signal in the mutants than in the WT at comparable stages. In addition to the delay in replication, the mutant lines exhibited an altered behaviour of DMC1 and RAD51 foci compared with Bowman. To confirm this, we followed the behaviour of HvRAD51 and HvDMC1 during synapsis using both TaASY1 and HvZYP1 antibodies (Figs 4, 5). We confirmed that in WT during leptotene HvDMC1 foci congregated towards one side of the nucleus, where the bouquet normally forms (Fig. 4A). This HvDMC1 localization is maintained at early zygotene (Fig. 4B); by mid-zygotene, HvDMC1 foci are dispersed within the nucleus, although some polarity is still retained around the ZYP1 axes (Fig. 4C). In the *des8* mutants, HvDMC1 foci were present but more diffuse in the nucleus and appeared to have lost their polarity at leptotene (Fig. 4D, G) and zygotene stages (Fig. 4E, H). By mid-zygotene, HvDMC1 still appeared more diffuse in the mutants compared with the WT (Fig. 4F, I).

Similarly, HvRAD51 foci were also present in BW248 (Fig. 5) but, unlike HvDMC1, they retained some polarity during early leptotene in both the WT (Fig. 5A) and the mutant (Fig. 5B). This HvRAD51 polarity was maintained at early zygotene in both the WT (Fig. 5C) and the mutant (Fig. 5D). At later zygotene, HvRAD51 retained some polarity in the WT (Fig. 5E), but foci were more diffuse in the mutant (Fig. 5F). Attempts were made to determine whether the number of HvDMC1 and HvRAD51 foci was also altered in the mutants, but high-count standard deviations meant that no significant differences were found (Supplementary Fig. S6).

### *des8.k* and *des8.l* both exhibit reduced crossovers

**Fig. 6.**
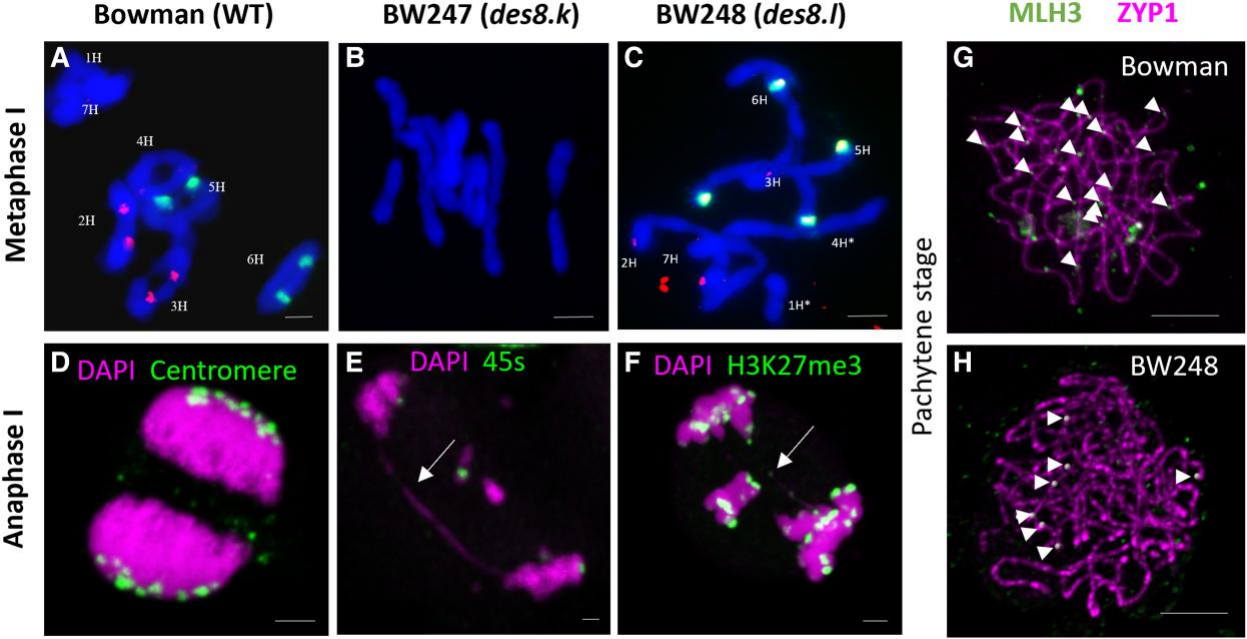
Metaphase and chromosome segregation. (A–C) Metaphase of Bowman (A), BW247 (B), and BW248 (C) stained with DAPI. Bowman (wild type; WT) (A) and BW248 (C) metaphase spreads were labelled with 45S rDNA (red) and 5S rDNA (green) probes. Asterisks indicate chromosomes with potential ambiguous labelling. (D–F) Chromosome segregation at Anaphase I in Bowman (D), BW247 (E), and BW248 (F), with chromatin labelled with DAPI (magenta) and centromeres (D), 45S rDNA (E), or H3K27me3 (F) labelled in green, showing abnormal division in both *HvXRCC2* mutants compared with the WT. Arrows in (E, F) indicate chromosome bridges. (G, H) Pachytene cells of Bowman (G) and BW248 (H) labelled with antibodies to HvZYP1 (magenta) and HvMLH3 (green). Arrowheads indicate 17 and 7 MLH3 foci on the HvZYP1 axes in Bowman (G) and BW248 (H), respectively. Scale bars=5 µm.

At Metaphase I, Bowman showed seven ring bivalents with a mean ±SD of 14.9±3 (*n*=50) chiasmata per cell (Fig. 6A; Supplementary Fig. S7A). BW247 and BW248 displayed an abnormal metaphase with a mean ±SD of 8.9 (±1.5, *n*=20) and 8.8 (±2.4, *n*=36) chiasmata, respectively (Fig. 6B, C; Supplementary Fig. S7A), representing a 40% reduction compared with the WT. The metaphase spreads also showed that, as previously reported in the original mutants, both BW247 and BW248 exhibit a range of ring and rod bivalents and univalents, indicating that some obligate crossovers (27%) had been lost (Supplementary Fig. S8). We used fluorescence *in situ* hybridization with probes for 45S and 5S rDNA to determine chiasmata frequencies of individual barley chromosomes of Bowman and BW248, and found that the number of chiasmata was reduced in all seven barley chromosomes in the mutant, particularly on chromosomes 4H and 6H (Supplementary Table S3). During Anaphase I, the centromeres pulled the homologous chromosomes equally to each side of the nucleus in Bowman (Fig. 6D), but both BW247 and BW248 showed lagging chromosomes, chromosome bridges (Fig. 6E, F, white arrows), and abnormal chromosome orientation (Fig. 6F, white arrow). Although finding pachytene cells was challenging, a comparison of MLH3-labelled foci marking class I crossovers was carried out in all genotypes. On average, we found 7.8 (±1.9, *n*=5), 7.1 (±1.4, *n*=20), and 17.2 (±2.3, *n*=14) MLH3 foci in BW247, BW248, and Bowman, respectively, again indicating a significant difference between the WT and the two mutant *des8* lines (Fig. 6G, H; Supplementary Fig. S7B).

### *des8.l* exhibits reduced recombination in genetic mapping

**Fig. 7.**
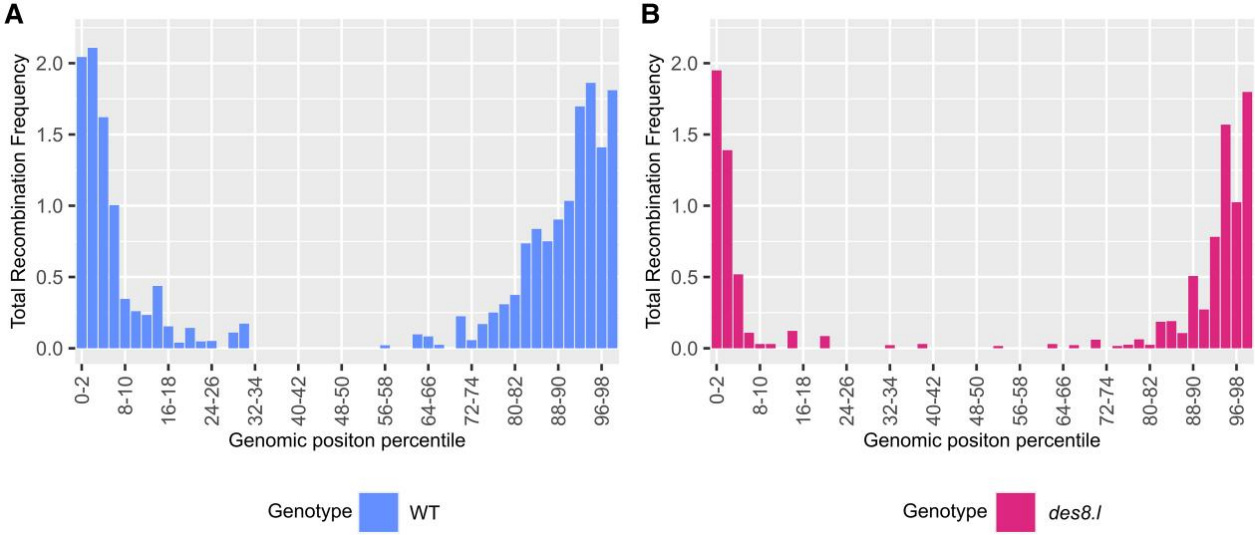
Comparison of genomic positions of recombination points in F_3_ families with wild-type (WT) and *des8.l* genotypes. Recombination found in F_3_ families derived from F_2_ individuals homozygous for either (A) WT (Bowman) or (B) *des8.l* (BW248) at *HvXRCC2*.

To study the change in recombination, F_3_ individuals from the BW248 (*des8.l*) × Morex cross were genotyped with the Barley 50K iSelect SNP array (Bayer *et al*., 2017). Our analysis utilized data from 11 916 polymorphic SNP loci on 82 F_3_ individuals from 10 homozygous *des8* WT F_2_ families and 78 F_3_ individuals from six homozygous *des8.l* F_2_ families. Across all chromosomes, there was a reduction in interstitial recombination frequency in *des8.l* compared with the WT (Fig. 7). The average number of crossovers observed was generally reduced across all chromosomes (20–68% reduction; Supplementary Table S4) with the exception of chromosome 3H, which showed almost no difference. This reduction in recombination reached statistical significance (*t*-test with Benjamini–Hochberg correction; Supplementary Table S4) in chromosomes 2H (*P*<0.0001), 4H (*P*<0.01), 5H (*P*<0.0001), and 6H (*P*=0.02) (Supplementary Fig. S9).

## Discussion

This study has shown that the semi-sterile *des8* mutants in barley are associated with either a deletion within or a deletion of the barley homolog of *XRCC2* (*HvXRCC2*) (Fig. 1). Effective null *xrcc2* mutants in the Chinese hamster cell line irs1 are sensitive to genotoxic agents (Mozdarani *et al*., 2001), and the potential sensitivity of *des8.l* to mitomycin C shown here (Supplementary Fig. S5) indicates that *XRCC2* potentially has a conserved role in DNA repair in barley, consistent with observations in both mammals and plants (Liu and Lim, 2005; Wang *et al*., 2014). However, it should be noted that the large deletion in *des8.l* spans seven high-confidence genes (Supplementary Table S1), so the role of *XRCC2* is unproven.

The two described *des8* alleles identified as spontaneous semi-sterile mutants were originally found in the cultivar Betzes (Hockett and Eslick, 1969), with both alleles (*des8.k* and *des8.l)* exhibiting similarly perturbed meiosis with univalents present at Metaphase I (Hernandez-Soriano, 1973). We found that these *Hvxrcc2* mutants initially exhibited normal meiotic progression, albeit with a small delay in initiation, with completion of synapsis (Fig. 2). However, the absence of *HvXRCC2* subsequently led to a dramatic reduction of crossovers, chromosome mis-segregation and semi-fertility, suggesting that *HvXRCC2* has a major role in recombination in barley (Fig. 6). The meiotic behaviour of both mutant alleles was very similar, with the potentially truncated allele *des8.k* showing similar disruption to meiotic progression (indeed, potentially displaying a greater delay) and ultimately the same level of reduction of crossovers as the knockout allele *des8.l* (Supplementary Fig. S7). The similarity of the phenotypes of the two *des8* mutants (truncated and knockout) highlights the potential importance of the C-terminal domain in the protein structure and in the interaction of Rad51 paralogs within the BCDX2 complex (Miller *et al*., 2004).

Both *des8* mutants exhibited a delay at the entry to meiosis, potentially related to XRCC2’s involvement in a genome ‘caretaker’ role and the resolution of DNA replication fork dynamics (Liu and Lim, 2005). Both *des8* mutants exhibited a delay in the initiation of synapsis and a noticeably more diffuse clustering of DMC1 foci compared with the WT (Fig. 3). This meiotic phenotype may relate to the known delay in (but not an absence of) RAD51 foci formation in *XRCC2* mutant hamster cells (Liu, 2002). The more diffuse DMC1 clustering may possibly reflect an issue in the recruitment of RAD51 and DMC1 in a timely manner (Pradillo *et al*., 2012; Da Ines *et al*., 2013b). Although differences in timing and distribution were noticed, no significant differences in the number of DMC1 foci were found, although this may be due to the inherent difficulties in such estimates (Supplementary Fig. S6). It is therefore possible that the final DMC1 foci number is maintained in the *des8* mutants but the dynamics of recruitment and retention may be compromised.

The observed reductions in crossover counts at Metaphase I and in recombination frequency shown in the genetic mapping of the *des8* mutants relative to the WT could relate directly to the known role of XRCC2 in HR (Liu *et al*., 1998; Masson *et al*., 2001; Liu, 2002). However, the reduction in proximal recombination found through genetic mapping in the *des8* mutant (Fig. 7) may also correspond to the observed delay at the beginning of meiosis and synapsis initiation. Such a change in distribution would align with the observed delay in synapsis and a subsequent disconnect with the spatiotemporal control of meiotic progression displayed by large-genome cereals such as barley (Higgins *et al*., 2012). It is likely that the skewed distribution of recombination found with the genetic mapping is potentially an underestimate of the effect of the *des8* mutant alleles at *HvXRCC2*, given that the mapping inherently selects against gametes resulting from chromosome mis-segregation that was evident cytologically (Fig. 6). In addition, the chiasmata counts from Metaphase I spreads may underestimate crossover numbers in the WT, as discussed previously (Colas *et al*., 2016), but are congruent with the MLH3 foci comparison between the mutants and the WT (Supplementary Fig. S7). Both experiments found an overall reduction in crossover/recombination frequency, with inter-chromosomal variation in the strength of this effect, although the reason for this variation in the effect of mutations in *HvXRCC2* is unclear.

The mutant phenotype displayed by *des8* barley plants is perhaps unexpected given the reported phenotypes shown by *XRCC2* mutants in other plant species. A recent study using virus-induced gene silencing to achieve knockdown of *XRCC2* in tetraploid wheat reported no associated change in the number of crossovers per chromosome but potentially some significant local effects in both pericentric and sub-telomeric regions (Raz *et al*., 2021). A more striking difference is to the phenotype reported in Arabidopsis, where the knockout *xrcc2* mutant is fully fertile and exhibits normal chromosome pairing, synapsis, and correct chromosome segregation (Bleuyard and White, 2004; Bleuyard *et al*., 2005; Da Ines *et al*., 2013a). Moreover, it shows an increased crossover rate and recombination (Da Ines *et al*., 2013a). Comparison between the two species is complicated by the different mutations underpinning the studies, with the Arabidopsis work based on a T-DNA insertion mutant (*atxrcc2–1*) with the T-DNA inserted in intron 5, 3 bp after the end of exon 5, that produces a truncated mRNA (Bleuyard *et al*., 2005). The Arabidopsis allele (*atxrcc2-1*) is thus potentially analogous to the barley *des8.k* (BW247) mutant, given the similar position of the truncation induced by the two mutation events (Supplementary Fig. S2). However, the meiotic phenotypes of these two analogous mutants are very different, implying that the gene has different meiotic roles in the two species. There are evident dissimilarities between the XRCC2 proteins of Arabidopsis and barley, and these differences may correspond to a dicot/monocot division (Supplementary Fig. S1). XRCC2 appears to be less well conserved than other RAD51 gene family members (Lin *et al*., 2006) and it is possible that this reflects divergent meiotic roles.

Despite the generally high conservation of the function of meiotic genes between organisms, significant differences in phenotype have previously been observed between plant species in meiotic mutant studies. For example, the effect of mutations in *MLH3* on synapsis in studies in barley compared with Arabidopsis (Jackson *et al*., 2006; Colas *et al*., 2016) and mutations in *ZYP1* homologs in rice compared with Arabidopsis or barley (Higgins *et al*., 2005; Wang *et al*., 2010; Barakate *et al*., 2014) are fundamentally different. Such comparisons are often complicated by the use of different mutagenesis strategies and the strength of the particular mutation events studied. However, the differences in mutant *XRCC2* phenotypes described here in barley compared with those previously described in Arabidopsis potentially indicate a fundamental difference in the roles the protein plays in meiosis in the two species.

The mutant phenotype displayed by *des8* barley plants is similar to the phenotype exhibited in mammalian systems, where *xrcc2* mutants are sterile, with clearly disturbed meiotic phenotypes alongside reduced recombination and aneuploidy (Cui *et al*., 1999; Griffin *et al*., 2000; Mozdarani *et al*., 2001). It should be noted, however, that the fertile mutant phenotype shown by Arabidopsis does align with that of Drosophila (Bayer *et al*., 2020). Given that the barley mutant phenotype for *xrcc2* matches the expectation derived from mammalian models, as is the case for *ZYP1* (Barakate *et al*., 2014), it is possible that, in these cases at least, model plant species with small genome sizes have the potential to display different mutant phenotypes reflecting potentially non-canonical roles for some meiotic genes. Alternatively, it may be that it is cereals such as barley that display atypical plant mutant phenotypes given the added complexities of their large genomes (Mascher *et al*., 2017; Lambing and Heckmann, 2018). In general, barley displays mutant phenotypes that are largely (but not always) aligned with expectations from model eukaryotic systems, and any changes in patterns of recombination occur within the skewed distribution of recombination associated with the spatial and temporal control of meiotic progression displayed by large-genome cereals (Higgins *et al*., 2012). The perturbed meiotic phenotype and reduction in recombination exhibited by the *des8* mutants in barley indicate that *XRCC2* is not a good candidate gene for practical exploitation to increase recombination frequency in crop plants, at least as a null mutant. *XRCC2* is thus unlike *FANCM* or *RecQ4*, which increase crossover counts in Arabidopsis and do show similar phenotypes in crop species including barley (Mieulet *et al*., 2018; Arrieta *et al*., 2021).

## Supplementary Material

eraf489_Supplementary_Data

## Data Availability

The data underlying this article are available upon request from the corresponding author. Scripts used in 50K recombination analysis are available at https://github.com/BioJNO/des8.

## Supplementary data

The following supplementary data are available at *JXB* online.

Fig. S1: Alignment of XRCC2 protein sequences of plant species.

Fig. S2: Comparison of Arabidopsis and barley XRCC2 protein sequences and positions of studied mutant events.

Fig. S3: Details of *des8.k* deletion in exon 6 of *HvXRCC2*.

Fig. S4: Expression levels of *HvXRCC2* shown in general and meiotic-specific expression datasets.

Fig. S5: Seedling root growth differences demonstrating mitomycin and bleomycin sensitivity.

Fig. S6: HvDMC1 and HvRAD51 foci counts at leptotene and zygotene for Bowman and *des8* mutants.

Fig. S7: Chiasmata and MLH3 foci counts for Bowman and *des8* mutants demonstrating reduction in the number of crossovers.

Fig. S8: Range of metaphase configurations shown by BW247 and BW248.

Fig. S9: Comparison of recombination in F_3_ lines derived from F_2_ lines homozygous for either WT or *des8.l*.

Table S1: Details of genes spanned by the deletion in BW248 (*des8.l*).

Table S2: Comparison of root lengths of Bowman, BW247, and BW248 grown in the absence or presence of genotoxic agents.

Table S3: Comparison of mean chiasmata frequency for each chromosome in Bowman and BW248.

Table S4: Comparison of crossover number for F_3_ lines derived from F_2_ lines homozygous for either WT *or des8.l.*

## References

[eraf489-B1] Adam J, Deans B, Thacker J. 2007. A role for Xrcc2 in the early stages of mouse development. DNA Repair 6, 224–234. 17116431 10.1016/j.dnarep.2006.10.024

[eraf489-B2] Arrieta M, Macaulay M, Colas I, Schreiber M, Shaw DP, Waugh R, Ramsay LD. 2021. An induced mutation in *HvRECQL4* increases overall recombination and restores fertility in a barley *HvMLH3* mutant background. Frontiers in Plant Science 12, 706560. 34868104 10.3389/fpls.2021.706560 PMC8633572

[eraf489-B3] Baker K, Dhillon T, Colas I, Cook N, Milne I, Milne L, Bayer M, Flavell AJ. 2015. Chromatin state analysis of the barley epigenome reveals a higher-order structure defined by H3K27me1 and H3K27me3 abundance. The Plant Journal 84, 111–124. 26255869 10.1111/tpj.12963 PMC4973852

[eraf489-B4] Barakate A, Higgins JD, Vivera S, et al 2014. The synaptonemal complex protein ZYP1 is required for imposition of meiotic crossovers in barley. The Plant Cell 26, 729–740. 24563202 10.1105/tpc.113.121269 PMC3967036

[eraf489-B5] Barakate A, Orr J, Schreiber M, et al 2021. Barley anther and meiocyte transcriptome dynamics in meiotic prophase I. Frontiers in Plant Science 11, 619404. 33510760 10.3389/fpls.2020.619404 PMC7835676

[eraf489-B6] Bayer FE, Deichsel S, Mahl P, Nagel AC. 2020. Drosophila Xrcc2 regulates DNA double-strand repair in somatic cells. DNA Repair 88, 102807. 32006716 10.1016/j.dnarep.2020.102807

[eraf489-B7] Bayer M, Rapazote-Flores P, Ganal M, et al 2017. Development and evaluation of a barley 50k iSelect SNP array. Frontiers in Plant Science 8, 1792. 29089957 10.3389/fpls.2017.01792 PMC5651081

[eraf489-B8] Bleuyard JY, White CI. 2004. The Arabidopsis homologue of Xrcc3 plays an essential role in meiosis. The EMBO Journal 23, 439–449. 14726957 10.1038/sj.emboj.7600055 PMC1271761

[eraf489-B9] Bleuyard JY, Gallego ME, Savigny F, White CI. 2005. Differing requirements for the Arabidopsis Rad51 paralogs in meiosis and DNA repair. The Plant Journal 41, 533–545. 15686518 10.1111/j.1365-313X.2004.02318.x

[eraf489-B10] Brown MS, Bishop DK. 2014. DNA strand exchange and RecA homologs in meiosis. Cold Spring Harbor Perspectives in Biology 7, a016659. 25475089 10.1101/cshperspect.a016659 PMC4292170

[eraf489-B11] Chun J, Buechelmaier ES, Powell SN. 2013. Rad51 paralog complexes BCDX2 and CX3 act at different stages in the BRCA1-BRCA2-dependent homologous recombination pathway. Molecular and Cell Biology 33, 387–395. 10.1128/MCB.00465-12 PMC3554112 23149936

[eraf489-B12] Close TJ, Bhat PR, Lonardi S, et al 2009. Development and implementation of high-throughput SNP genotyping in barley. BMC Genomics 10, 582. 19961604 10.1186/1471-2164-10-582 PMC2797026

[eraf489-B13] Colas I, Macaulay M, Higgins JD, et al 2016. A spontaneous mutation in MutL-Homolog 3 (HvMLH3) affects synapsis and crossover resolution in the barley desynaptic mutant *des10*. New Phytologist 212, 693–707. 27392293 10.1111/nph.14061

[eraf489-B14] Colas I, Darrier B, Arrieta M, Mittmann SU, Ramsay L, Sourdille P, Waugh R. 2017. Observation of extensive chromosome axis remodelling during the “diffuse-phase” of meiosis in large genome cereals. Frontiers in Plant Science 8, 1235. 28751906 10.3389/fpls.2017.01235 PMC5508023

[eraf489-B15] Colas I, Barakate A, Macaulay M, Schreiber M, Stephens J, Vivera S, Halpin C, Waugh R, Ramsay L. 2019. *desynaptic5* carries a spontaneous semi-dominant mutation affecting *Disrupted Meiotic cDNA 1* in barley. Journal of Experimental Botany 70, 2683–2698. 31028386 10.1093/jxb/erz080 PMC6509107

[eraf489-B16] Cui X, Brenneman M, Meyne J, Oshimura M, Goodwin EH, Chen DJ. 1999. The *XRCC2* and *XRCC3* repair genes are required for chromosome stability in mammalian cells. Mutation Research 434, 75–88. 10422536 10.1016/s0921-8777(99)00010-5

[eraf489-B17] Da Ines O, Abe K, Goubely C, Gallego ME, White CI. 2012. Differing requirements for RAD51 and DMC1 in meiotic pairing of centromeres and chromosome arms in *Arabidopsis thaliana*. PLoS Genetics 8, 245–256. 10.1371/journal.pgen.1002636 PMC3330102 22532804

[eraf489-B18] Da Ines O, Degroote F, Amiard S, Goubely C, Gallego ME, White CI. 2013a. Effects of *XRCC2* and *RAD51B* mutations on somatic and meiotic recombination in *Arabidopsis thaliana*. The Plant Journal 74, 959–970. 23521529 10.1111/tpj.12182

[eraf489-B19] Da Ines O, Degroote F, Goubely C, Amiard S, Gallego ME, White CI. 2013b. Meiotic recombination in Arabidopsis is catalysed by DMC1, with RAD51 playing a supporting role. PLoS Genetics 9, e1003787. 24086145 10.1371/journal.pgen.1003787 PMC3784562

[eraf489-B20] Daley JM, Gaines WA, Kwon Y, Sung P. 2014. Regulation of DNA pairing in homologous recombination. Cold Spring Harbor Perspectives in Biology 6, a017954. 25190078 10.1101/cshperspect.a017954 PMC4413238

[eraf489-B21] Druka A, Franckowiak J, Lundqvist U, et al 2010. Exploiting induced variation to dissect quantitative traits in barley. Biochemical Society Transaction 38, 683–688. 10.1042/BST0380683 20298243

[eraf489-B22] Druka A, Franckowiak J, Lundqvist U, et al 2011. Genetic dissection of barley morphology and development. Plant Physiology 155, 617–627. 21088227 10.1104/pp.110.166249 PMC3032454

[eraf489-B23] Griffin CS, Simpson PJ, Wilson CR, Thacker J. 2000. Mammalian recombination-repair genes *XRCC2* and *XRCC3* promote correct chromosome segregation. Nature Cell Biology 2, 757–761. 11025669 10.1038/35036399

[eraf489-B24] Hernandez-Soriano JM . 1973. Desynaptic mutants in Betzes barley. MSc Thesis, The University of Arizona.

[eraf489-B25] Higgins JD, Sanchez-Moran E, Armstrong SJ, Jones GH, Franklin FC. 2005. The *Arabidopsis* synaptonemal complex protein ZYP1 is required for chromosome synapsis and normal fidelity of crossing over. Genes & Development 19, 2488–2500. 16230536 10.1101/gad.354705 PMC1257403

[eraf489-B26] Higgins JD, Perry RM, Barakate A, Ramsay L, Waugh R, Halpin C, Armstrong SJ, Franklin FC. 2012. Spatiotemporal asymmetry of the meiotic program underlies the predominantly distal distribution of meiotic crossovers in barley. The Plant Cell 24, 4096–4109. 23104831 10.1105/tpc.112.102483 PMC3517238

[eraf489-B27] Hockett EA, Eslick RF. 1969. Spontaneous frequencies of genetic and other sterilities in barley *Hordeum vulgare* L. Crop Science 9, 23–24.

[eraf489-B28] Jackson N, Sanchez-Moran E, Buckling E, Armstrong SJ, Jones GH, Franklin FC. 2006. Reduced meiotic crossovers and delayed prophase I progression in AtMLH3-deficient *Arabidopsis*. The EMBO Journal 25, 1315–1323. 16467846 10.1038/sj.emboj.7600992 PMC1422170

[eraf489-B29] Jasencakova Z, Meister A, Schubert I. 2001. Chromatin organization and its relation to replication and histone acetylation during the cell cycle in barley. Chromosoma 110, 83–92. 11453558 10.1007/s004120100132

[eraf489-B30] Künzel G, Waugh R. 2002. Integration of microsatellite markers into the translocation-based physical RFLP map of barley chromosome 3H. Theoretical and Applied Genetics 105, 660–665. 12582478 10.1007/s00122-002-0913-5

[eraf489-B31] Lambing C, Heckmann S. 2018. Tackling plant meiosis: from model research to crop improvement. Frontiers in Plant Science 9, 829. 29971082 10.3389/fpls.2018.00829 PMC6018109

[eraf489-B32] Lin Z, Kong H, Nei M, Ma H. 2006. Origins and evolution of the *recA*/*RAD51* gene family: evidence for ancient gene duplication and endosymbiotic gene transfer. Proceedings of the National Academy of Sciences, USA 103, 10328–10333. 10.1073/pnas.0604232103 PMC1502457 16798872

[eraf489-B33] Liu N, Lamerdin JE, Tebbs RS, et al 1998. XRCC2 and XRCC3, new human Rad51-family members, promote chromosome stability and protect against DNA cross-links and other damages. Molecular Cell 1, 783–793. 9660962 10.1016/s1097-2765(00)80078-7

[eraf489-B34] Liu N . 2002. XRCC2 is required for the formation of Rad51 foci induced by ionizing radiation and DNA cross-linking agent mitomycin C. BioMed Research International 2, 106–113. 10.1155/S1110724302204040 PMC153789 12488590

[eraf489-B35] Liu N, Lim CS. 2005. Differential roles of XRCC2 in homologous recombinational repair of stalled replication forks. Journal of Cellular Biochemistry 95, 942–954. 15861395 10.1002/jcb.20457

[eraf489-B36] Lundqvist U, Franckowiak JD, Konishi T. 1997. New and revised descriptions of barley genes. Barley Genetics Newsletter 26, 22–516.

[eraf489-B37] Mascher M, Gundlach H, Himmelbach A, et al 2017. A chromosome conformation capture ordered sequence of the barley genome. Nature 544, 427–433. 28447635 10.1038/nature22043

[eraf489-B38] Masson JY, Tarsounas MC, Stasiak AZ, Stasiak A, Shah R, McIlwraith MJ, Benson FE, West SC. 2001. Identification and purification of two distinct complexes containing the five RAD51 paralogs. Genes & Development 15, 3296–3307. 11751635 10.1101/gad.947001 PMC312846

[eraf489-B39] Mercier R, Mézard C, Jenczewski E, Macaisne N, Grelon M. 2014. The molecular biology of meiosis in plants. Annual Reviews in Plant Biology 66, 297–327. 10.1146/annurev-arplant-050213-035923 25494464

[eraf489-B40] Mieulet D, Aubert G, Bres C, et al 2018. Unleashing meiotic crossovers in crops. Nature Plants 4, 1010–1016. 30478361 10.1038/s41477-018-0311-x

[eraf489-B41] Miller KA, Sawicka D, Barsky D, Albala JS. 2004. Domain mapping of the Rad51 paralog protein complexes. Nucleic Acids Research 32, 169–178. 14704354 10.1093/nar/gkg925 PMC373258

[eraf489-B42] Milne L, Bayer M, Rapazote-Flores P, Mayer CD, Waugh R, Simpson CG. 2021. EORNA, a barley gene and transcript abundance database. Science Data 8, 90. 10.1038/s41597-021-00872-4 PMC7994555 33767193

[eraf489-B43] Mozdarani H, Liu N, Jones NJ, Bryant PE. 2001. The XRCC2 human repair gene influences recombinational rearrangements leading to chromatid breaks. International Journal of Radiation Biology 77, 859–865. 11571019 10.1080/09553000110054890

[eraf489-B44] Pradillo M, López E, Linacero R, Romero C, Cuñado N, Sánchez-Morán E, Santos JL. 2012. Together yes, but not coupled: new insights into the roles of RAD51 and DMC1 in plant meiotic recombination. The Plant Journal 69, 921–933. 22066484 10.1111/j.1365-313X.2011.04845.x

[eraf489-B45] Rapazote-Flores P, Bayer M, Milne L, et al 2019. BaRTv1.0: an improved barley reference transcript dataset to determine accurate changes in the barley transcriptome using RNA-seq. BMC Genomics 20, 968. 31829136 10.1186/s12864-019-6243-7 PMC6907147

[eraf489-B46] Raz A, Dahan-Meir T, Melamed-Bessudo C, Leshkowitz D, Levy AA. 2021. Redistribution of meiotic crossovers along wheat chromosomes by virus-induced gene silencing. Frontiers in Plant Sciences 11, 635139. 10.3389/fpls.2020.635139 PMC7890124 33613593

[eraf489-B47] Saxena S, Somyajit K, Nagaraju G. 2018. XRCC2 regulates replication fork progression during dNTP alterations. Cell Reports 25, 3273–3282.e6. 30566856 10.1016/j.celrep.2018.11.085

[eraf489-B48] Sullivan MR, Bernstein KA. 2018. RAD-ical new insights into RAD51 regulation. Genes 9, 629. 30551670 10.3390/genes9120629 PMC6316741

[eraf489-B49] Wang M, Wang K, Tang D, Wei C, Li M, Shen Y, Chi Z, Gu M, Cheng Z. 2010. The central element protein ZEP1 of the synaptonemal complex regulates the number of crossovers during meiosis in rice. The Plant Cell 22, 417–430. 20154151 10.1105/tpc.109.070789 PMC2845403

[eraf489-B50] Wang Y, Xiao R, Wang H, Cheng Z, Li W, Zhu G, Wang Y, Ma H. 2014. The Arabidopsis *RAD51* paralogs *RAD51B*, *RAD51D* and *XRCC2* play partially redundant roles in somatic DNA repair and gene regulation. New Phytologist 201, 292–304. 24102485 10.1111/nph.12498

[eraf489-B51] Wang Y, Copenhaver GP. 2018. Meiotic recombination: mixing it up in plants. Annual Review of Plant Biology 69, 577–609. 10.1146/annurev-arplant-042817-040431 29489392

[eraf489-B52] Yang Y, Guo J, Dai L, et al 2018. XRCC2 mutation causes meiotic arrest, azoospermia and infertility. Journal of Medical Genetics 55, 628–636. 30042186 10.1136/jmedgenet-2017-105145 PMC6119352

[eraf489-B53] Zickler D . 2006. From early homologue recognition to synaptonemal complex formation. Chromosoma 115, 158–174. 16570189 10.1007/s00412-006-0048-6

