# Supplementary material for: The RAD51 paralogue HvXRCC2 affects meiosis and recombination in barley
Source: J Exp Bot. 2025 Dec 18;77(12):3593–606. doi: 10.1093/jxb/eraf489 (PMC13293090; doi:10.1093/jxb/eraf489)
Supplement: eraf489_Supplementary_Data [file eraf489_supplementary_data.pdf]

Figure S1: Alignment of XRCC2 protein sequences of plant species

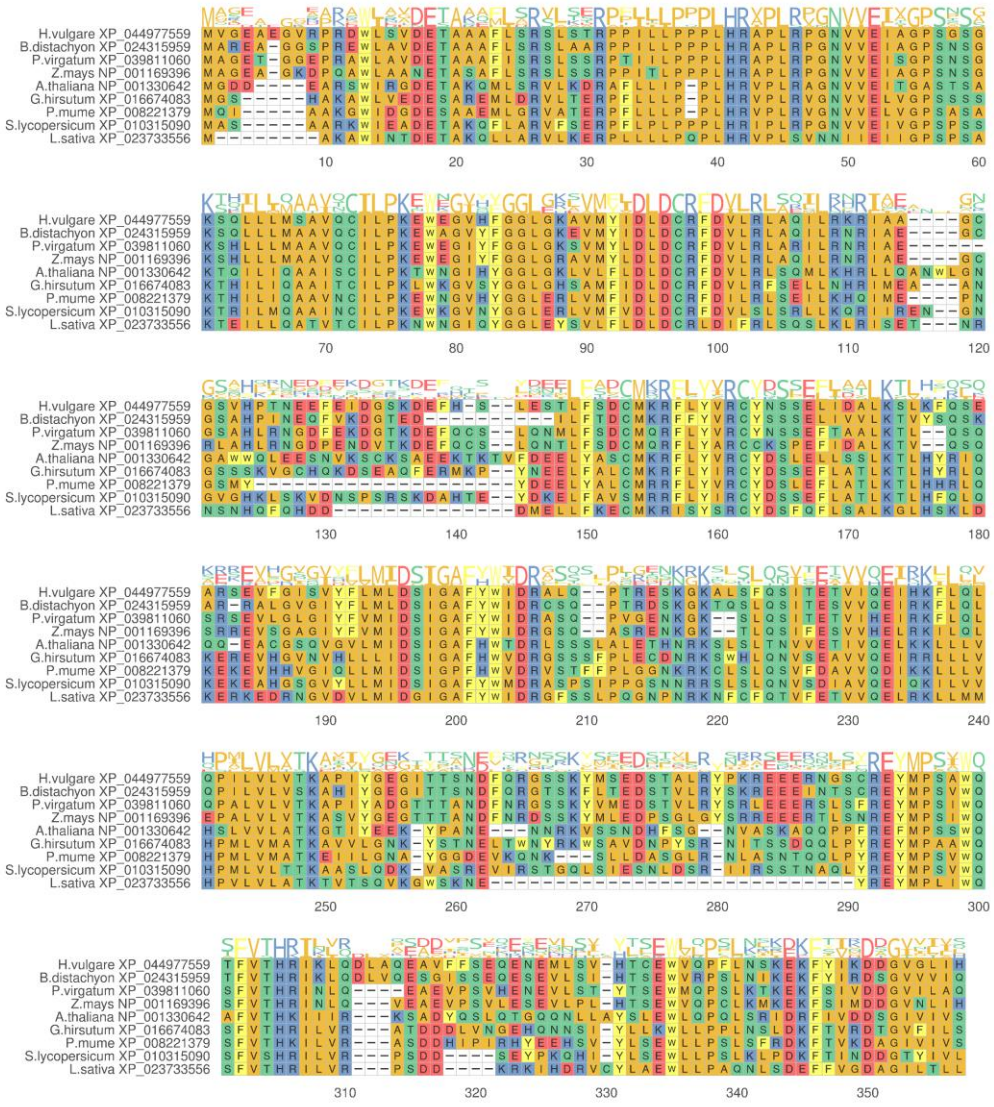

Multiple sequence alignment produced using ggplot2 and visualised with ggmsa

Figure S2: Comparison of Arabidopsis and barley XRCC2 protein sequences and positions of studied mutant events

Protein sequence comparison of AtXRCC2 v HvXRCC2 showing the conservation of Walker A and Walker B domains and the positions of the deletions in *atxrcc2-1* in Arabidopsis and *des8.k* in barley.

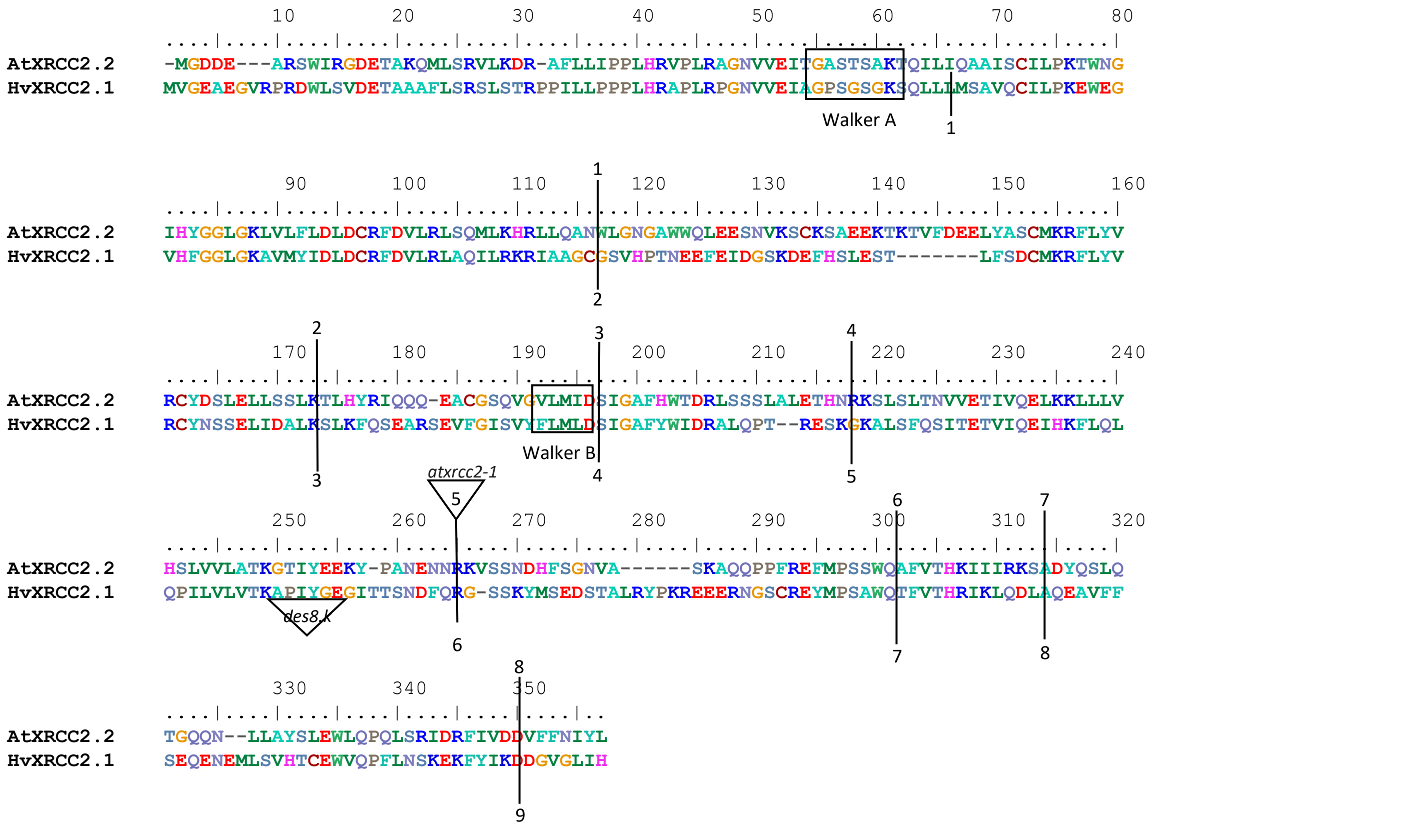

Figure S3: Details of *des8.k* deletion in exon six of *HvXRCC2*.

Bowman (wt)

```
             K  A  L  S  F  Q  S  I  T  E  T  V  I  Q  E  I  H  K  F  L  Q  L  Q
cctgtctttgcagGAAAGCTCTATCATTTCAGAGTATCACTGAAACTGTTATCCAGGAGATACACAAGTTTTTGCAACTTCAAC

P  I  L  V  L  V  T  K  A  P  I  Y  G  E  G  I  T  T  S  N  D  F  Q  R
CTATTTTGGTGTTGGTCACAAAGGCACCTATTTATGGTGAAGGAATCACAACATCAAACGACTTCCAAAGgtaatccatga...

                            G  S  S  K  Y  M  S  E  D  S  T  A  L  R  Y  P  K  R
...atattgtctctttaatggtatttagGGGTTCTTCAAAATACATGTCAGAGGATTCAACAGCTTTGAGATATCCGAAACGG
```

BW247 (*des8.k*)

```
             K  A  L  S  F  Q  S  I  T  E  T  V  I  Q  E  I  H  K  F  L  Q  L  Q
cctgtctttgcagGAAAGCTCTATCATTTCAGAGTATCACTGAAACTGTTATCCAGGAGATACACAAGTTTTTGCAACTTCAAC

P  I  L  V  L  V  T  K  E  S  Q  H  Q  T  T  S  K
CTATTTTGGTGTTGGTCACAAAGGAATCACAACATCAAACGACTTCCAAAGgtaatccatgatctgttatcactaaacact...

                           G  V  L  Q  N  T  C  Q  R  I  Q  Q  L  *
...atattgtctctttaatggtatttagGGGTTCTTCAAAATACATGTCAGAGGATTCAACAGCTTTGAGATATCCGAAACGGG
```

19 bp deletion (red) causes frameshift within conserved ATP binding site resulting in a putative truncated protein (347 aa > 262 aa)

Figure S4: Expression levels of *HvXRCC2* shown in general and meiotic specific expression datasets.

A- An expression atlas across 16 tissues of cv. Morex (Rapazote-Flores et al 2019) (EMB=Embryo, ROO=roots (10cm shoot stage), ROO2=roots(28 DAP), LEA=leaf (10cm shoot stage), NOD=developing tillers 3rd internode(42 DAP), INF1=young developing inflorescences(5mm), INF2= developing inflorescences (1-1.5cm), CAR5=developing grain (5DAP), CAR15=developing grain (15DAP), ETI=etiolated seedling in dark conditions (10DAP), LEM=lemma (42DAP), LOD=lodicule (42DAP), PAL=palea (42DAP), RAC=rachis(35DAP), EPI=epidermal strips (28DAP), SEN=senescing leaves (56DAP).
B- A meiotic specific expression dataset from cv. Golden Promise (Barakate et al 2020) (A.Pre=pre-meiotic anthers, A.LepZyg= anthers at leptotene/zygotene, A.PacDip= anthers at pachytene/diplotene, A.MetTet= anthers at Metaphase-Tetrad, M. LepZyg= meiocytes at leptotene/zygotene, M.PacDip= meiocytes at pachytene/diplotene, GEmb=embryo from mature grain. *HvXRCC2* exists in two main isoforms, BAnTr.GP.3HG012678.1 (blue) and BAnTr.GP.3HG012678.2 (green) which has an alternative 5' splice site of 3 nucleotides at the end of exon 2 but is generally expressed at a lower level.

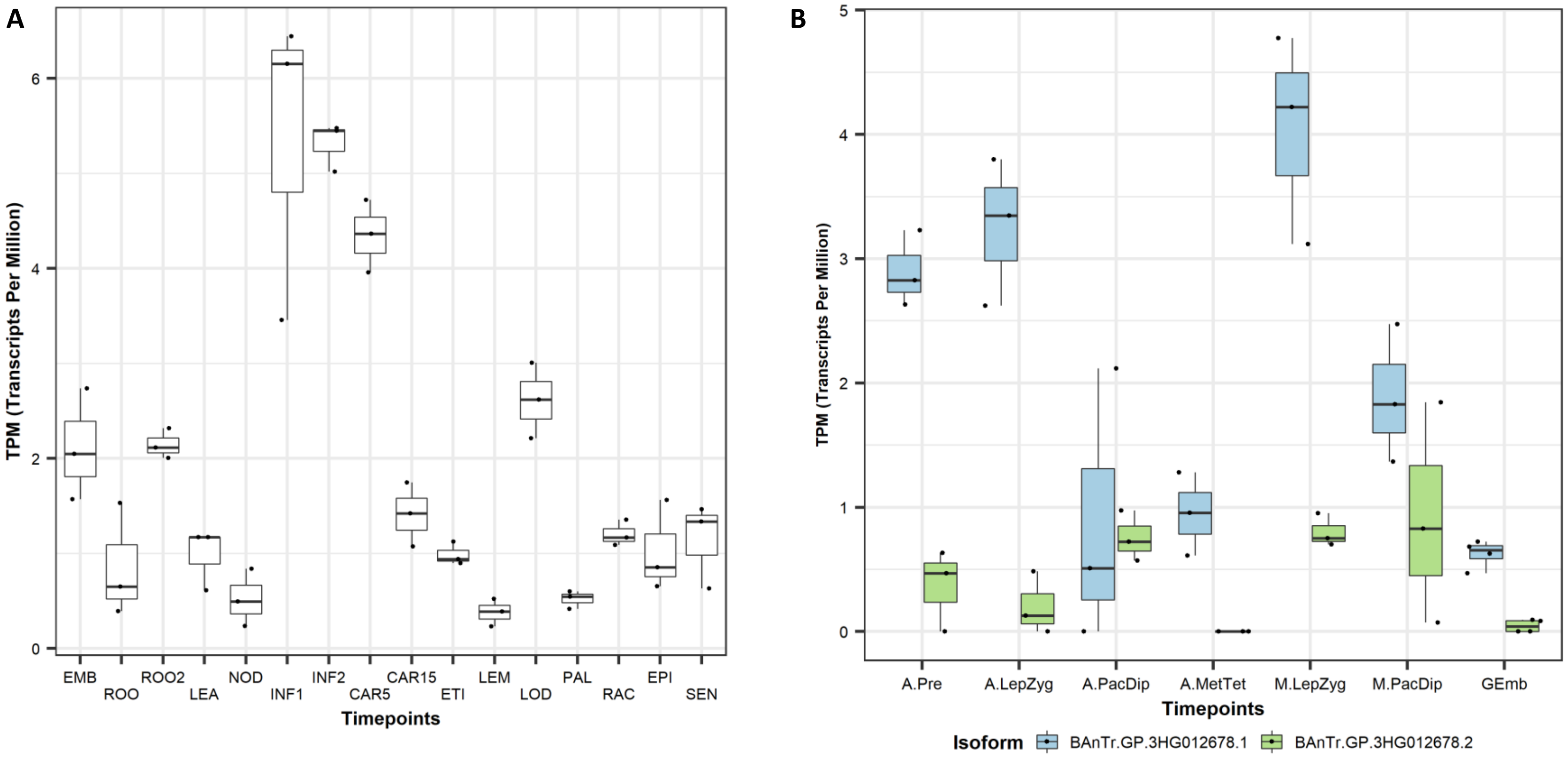

Figure S5: Seedling root growth differences demonstrating Mitomycin and Bleomycin sensitivity

A- Slow root growth for all genotypes with no significant difference found between WT and *des8* when exposed to bleomycin (which causes extra DSBs)
B- Longer roots in WT compared to *des8* mutants when exposed to Mitomycin (DNA inter-strand crosslinking agent)

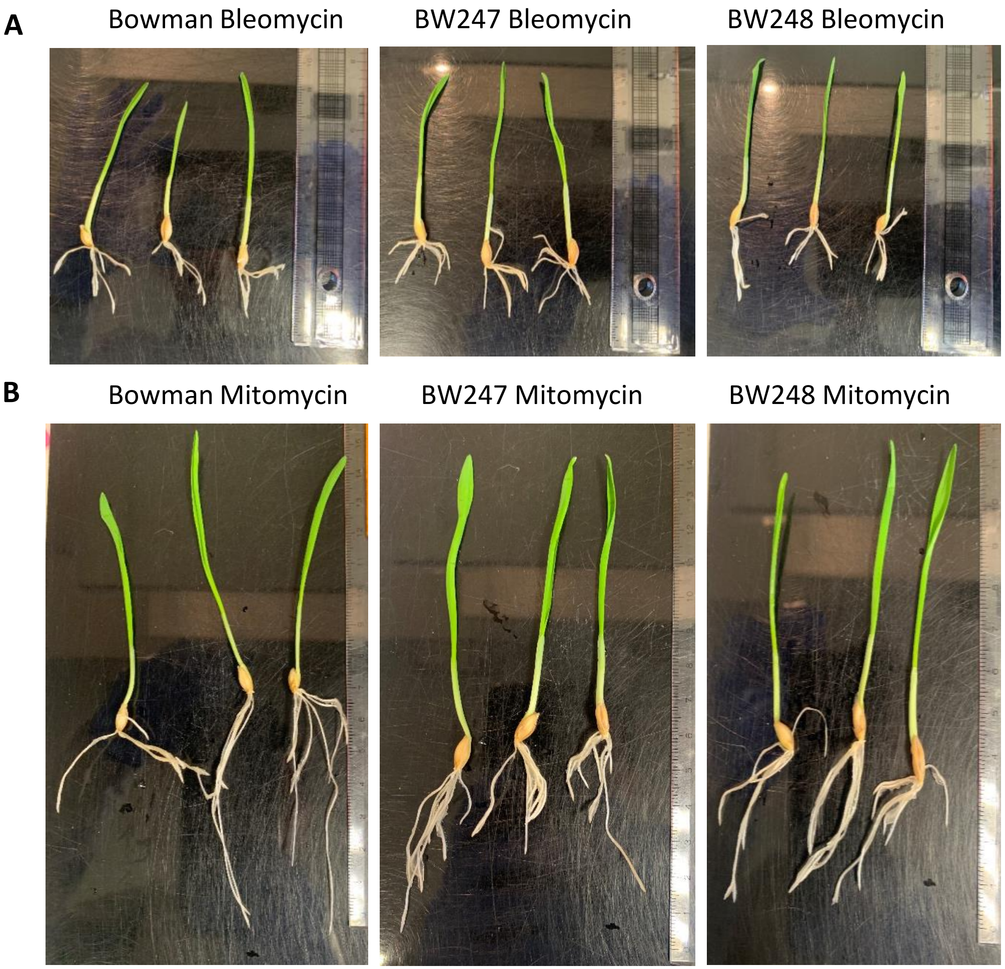

Figure S6: HvDMC1 and HvRAD51 foci counts at leptotene and zygotene for Bowman and *des8* mutants

A- DMC1 foci number increased from leptotene to zygotene in all genotypes. At leptotene, BW247 and BW248 had 258.4± 37.7 (n=17) and 273.5± 61.8 (n=19) respectively while Bowman displayed 199.4± 45.8 (n=20). At early zygotene, BW247 and BW248 showed 413.1±112.6 (n=10) and 344.2± 85.2 (n=17) foci respectively while Bowman had 390.9± 75.0 (n=17). At mid zygotene BW247 and BW248 had 324.9±72.7 (n=115) and 356.7± 99.6 (n=22) foci respectively while Bowman had 407.4±79.3 (n=14).
B- RAD51 foci again showed an increase from leptotene to zygotene for both Bowman (WT) and BW248 (*des8.l*). However, estimation of foci number was even more difficult than for DMC1 due to the foci being maintained in a tight cluster.

.

A- DMC1 foci

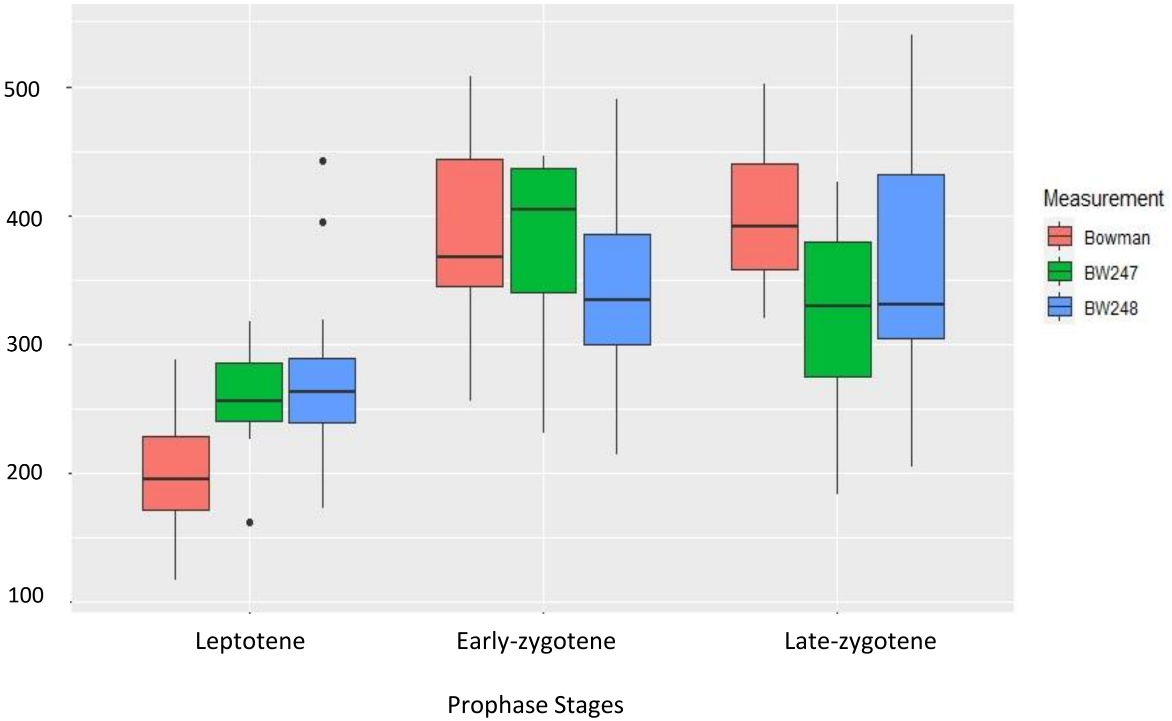

B- RAD51 foci

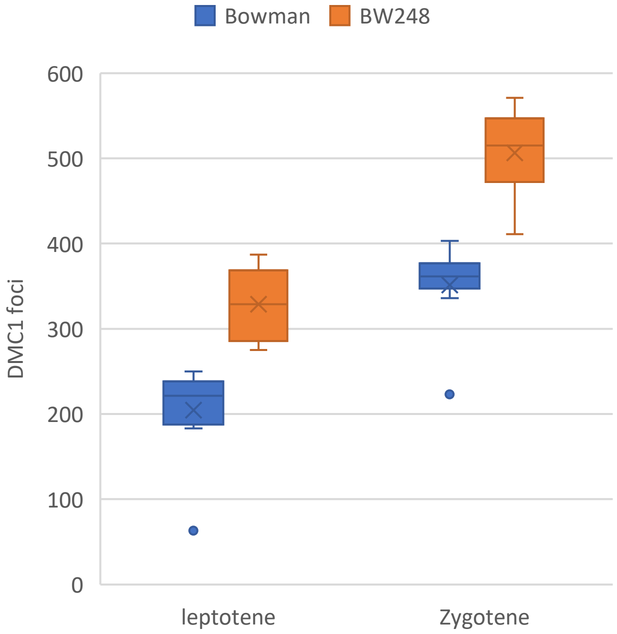

Figure S7: Chiasmata and MLH3 foci counts for Bowman and *des8* mutants demonstrating reduction in number of crossovers.
A- Boxplots of number of chiasmata from Metaphase I spreads (in red) and the number of ring bivalents (green), rod bivalents (cyan) and univalent (purple) for Bowman (WT), BW247 (*des8.k*) and BW248 (*des8.l*).
B- Boxplots of the MHvMLH3 foci counts at pachytene for Bowman (WT), BW247 (*des8.k*) and BW248 (*des8.l*).

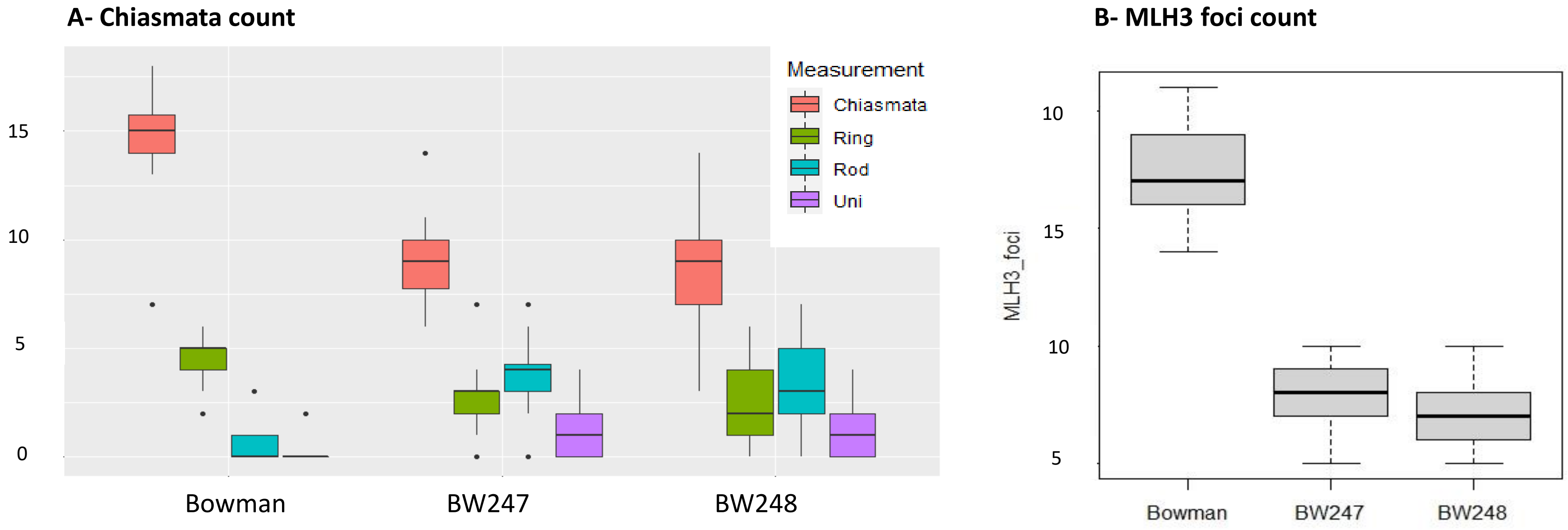

Figure S8: Range of Metaphase configurations shown by BW247 and BW248.

Metaphase spreads show a mixture of metaphase configuration in both (A)BW247 (*des8.k)* and (B) BW248 *(des8.l)* with a range shown of ring bivalents, rod-bivalents and univalents, indicative of loss of obligate crossovers. Scale bar 5µm

A- BW247

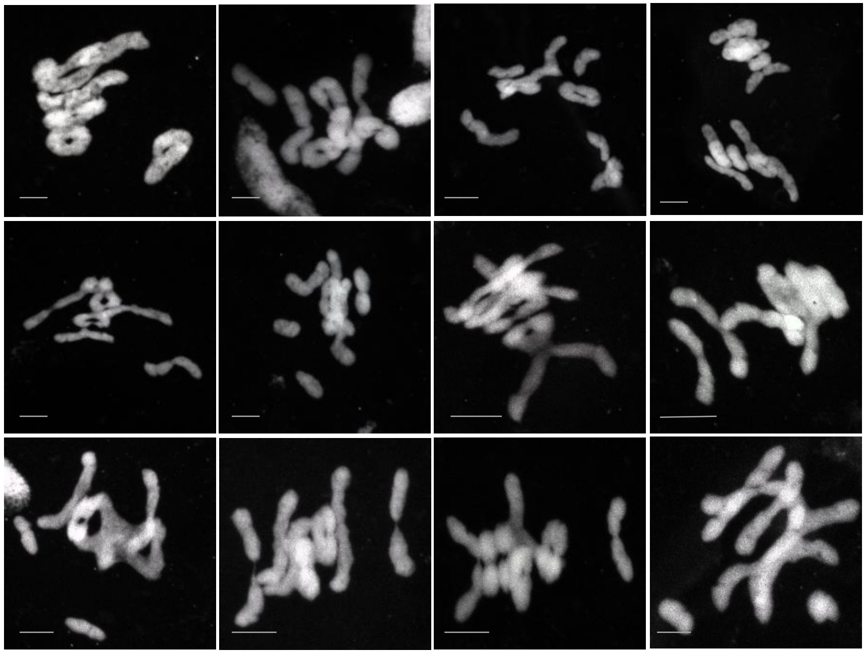

B- BW248

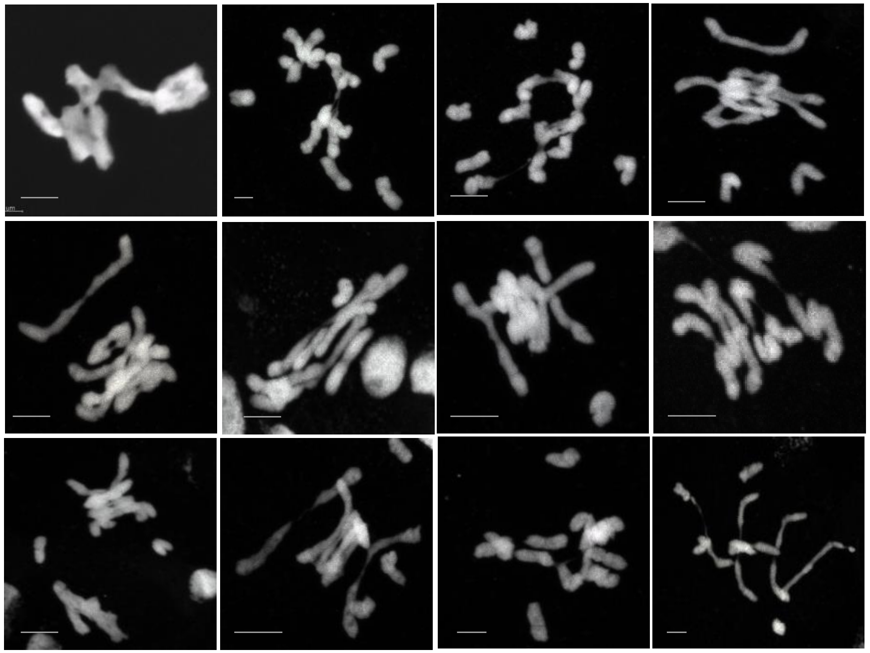

Figure S9: **Comparison of recombination in F_3 lines derived from F_2 lines homozygous for either WT or *des8.l*.**

A. Plots of genetic versus physical position for mapped SNPs from 50K chip for F_3 populations derived from F_2 individuals homozygous for either WT (blue) or *des8.l* (yellow) at *Hvxrcc2.* The x axes are shaded into the three genomic zones designated in Mascher et al. (2017).

B. Genetic length of each chromosome from map derived from F_3 populations derived from F_2 individuals homozygous for either WT (blue) or *des8.l* (pink) at *Hvxrcc2*

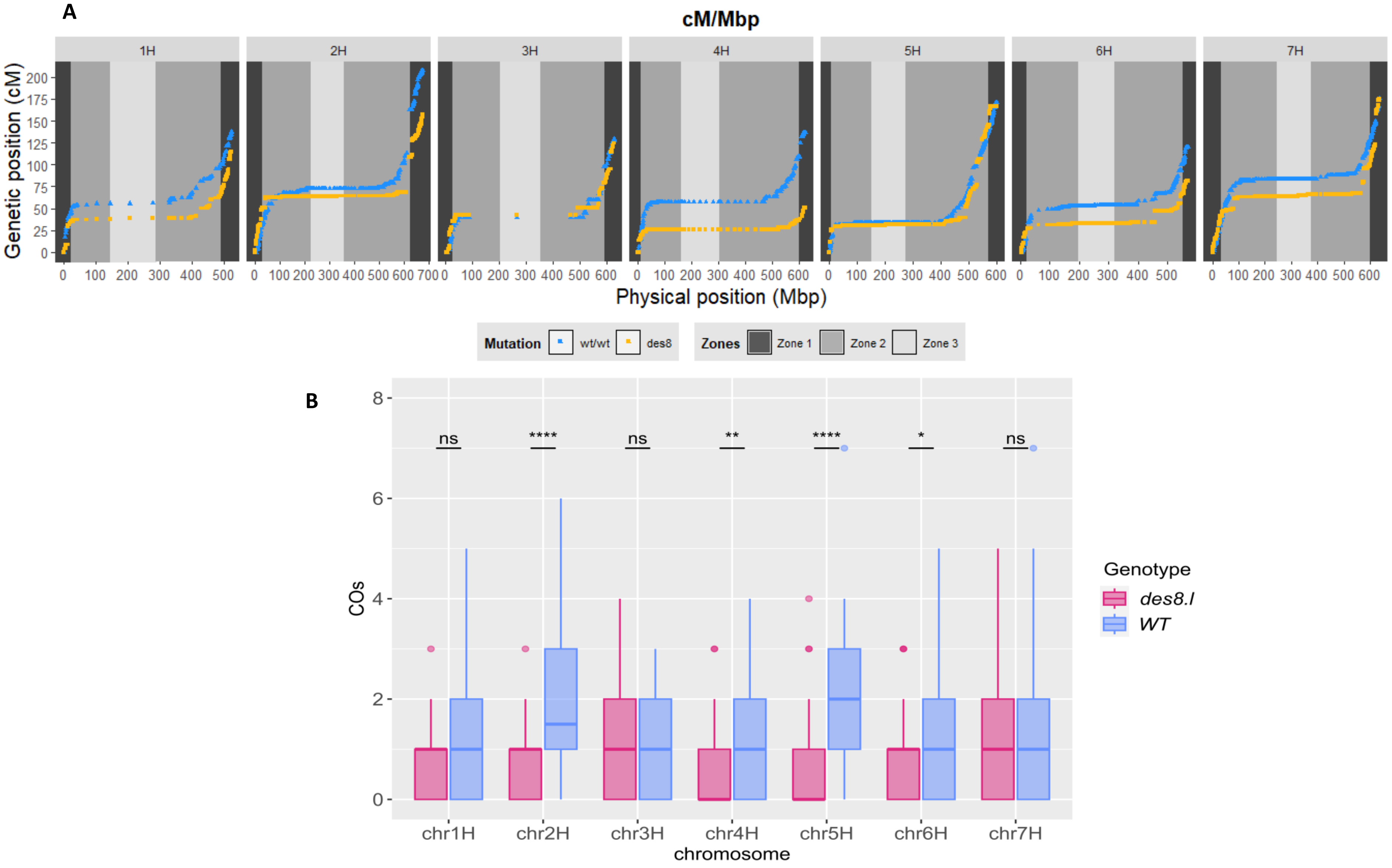

Table S1: Details of genes spanned by the deletion in BW248 (*des8.I*).

Chromosome	start	end	orientation	ID	confidence	description
chr3H	535746452	535752114	-	HORVU.MOREX.r3.3HG0299140.1	high	DNA repair protein
chr3H	535840752	535841874	-	HORVU.MOREX.r3.3HG0299150.1	high	Pectinesterase inhibitor, putative
chr3H	535879735	535881671	-	HORVU.MOREX.r3.3HG0299160.1	high	Hexosyltransferase
chr3H	535932498	535934514	+	HORVU.MOREX.r3.3HG0299170.1	low	Zinc-binding dehydrogenase family protein
chr3H	535950797	535951246	+	HORVU.MOREX.r3.3HG0299180.1	low	RNA-directed DNA polymerase (Reverse transcriptase)
chr3H	535952123	535952584	+	HORVU.MOREX.r3.3HG0299190.1	low	Retrotransposon protein, putative, unclassified
chr3H	535957563	535962464	+	HORVU.MOREX.r3.3HG0299200.1	high	Ion channel DMI1
chr3H	535963698	535964132	-	HORVU.MOREX.r3.3HG0299210.1	low	Glutathione S-transferase T3
chr3H	535966213	535970570	-	HORVU.MOREX.r3.3HG0299220.1	high	Kinase family protein
chr3H	536195400	536198325	-	HORVU.MOREX.r3.3HG0299230.1	low	Chlorophyll a-b binding protein, chloroplastic
chr3H	536200118	536221226	-	HORVU.MOREX.r3.3HG0299240.1	high	TLC domain-containing protein 2
chr3H	536221333	536224534	+	HORVU.MOREX.r3.3HG0299250.1	high	Nuclear matrix protein 1

N.B. position and annotation taken from Morex genomeV3 (https://plants.ensembl.org/Hordeum_vulgare/Info/Index)

Table S2: Comparison of root lengths of Bowman, BW247 and BW248 grown in the absence or presence of genotoxic agents. Longest root measured in mm. of seven-day old seedlings

	**Control**			**Bleomycin**			**Mitomycin**		
	Mean	SE	n	Mean	SE	n	Mean	SE	n
**Bowman**	65.4	2.6	14	13.8	1.1	16	24.7	1.7	16
**BW247 (*des8.k*)**	62.9	1.4	17	12.1	1.4	17	26.2	1.8	17
**BW248 (*des8.l*)**	67.9	3.9	14	12.2	1.4	16	20.0	1.4	16

Table S3: Comparison of mean chiasmata frequency for each chromosome in Bowman and BW248.

Comparison of mean chiasmata frequency per chromosome in Bowman and BW248
N.B. The low count in 6H may be influenced by the difficulty in distinguishing chromosomes 5H and 6H

Chromosomes	Bowman	BW248	CO Reduction %	% Univalents
1H	2.00	1.26	37.0	6.0
2H	2.08	1.50	27.9	0.0
3H	2.52	1.46	42.1	11.6
4H	2.22	0.88	60.4	60.0
5H	2.12	1.06	50.0	31.6
6H	1.92	0.36	81.3	64.0
7H	2.08	1.14	45.2	16.7

Table S4: Comparison of crossover number for F_3 lines derived from F_2 lines homozygous for either WT or *des8.l*

Chromosome	WT CO	*des8.l* CO	% change	p	p.adj	p.format	p.signif
1H	91	61	-33.00%	0.047278	0.066	0.0473	ns
2H	143	57	-60.10%	6.15E-08	2.2E-07	6.1E-08	****
3H	86	82	-4.70%	0.986313	0.99	0.9863	ns
4H	87	48	-44.80%	0.003737	0.0087	0.0037	**
5H	148	48	-67.60%	4.62E-10	3.2E-09	4.60E-10	****
6H	100	63	-37.00%	0.015648	0.027	0.0156	*
7H	119	95	-20.20%	0.267609	0.31	0.2676	ns
